# Chromatin Remodeling of Colorectal Cancer Liver Metastasis is Mediated by an HGF‐PU.1‐DPP4 Axis

**DOI:** 10.1002/advs.202004673

**Published:** 2021-08-10

**Authors:** Lihua Wang, Ergang Wang, Jorge Prado Balcazar, Zhenzhen Wu, Kun Xiang, Yi Wang, Qiang Huang, Marcos Negrete, Kai‐Yuan Chen, Wei Li, Yujie Fu, Anders Dohlman, Robert Mines, Liwen Zhang, Yoshihiko Kobayashi, Tianyi Chen, Guizhi Shi, John Paul Shen, Scott Kopetz, Purushothama Rao Tata, Victor Moreno, Charles Gersbach, Gregory Crawford, David Hsu, Emina Huang, Pengcheng Bu, Xiling Shen

**Affiliations:** ^1^ Department of Biomedical Engineering Duke University Durham NC 27708 USA; ^2^ Key Laboratory of RNA Biology Key Laboratory of Protein and Peptide Pharmaceutical Institute of Biophysics Chinese Academy of Sciences Beijing 100101 China; ^3^ University of Chinese Academy of Sciences Beijing 100049 China; ^4^ Department of Cell Biology Regeneration Next Duke University School of Medicine Durham NC 27710 USA; ^5^ Laboratory Animal Research Center Institute of Biophysics Chinese Academy of Sciences Beijing 100101 China; ^6^ Department of Gastrointestinal Medical Oncology MD Anderson Durham NC 77030 USA; ^7^ Department of Clinical Sciences University of Barcelona Barcelona 08193 Spain; ^8^ Prevention and Control Program Catalan Institute of Oncology‐IDIBELL CIBERESP Barcelona E08907 Spain; ^9^ Department of Pediatrics Duke University School of Medicine Durham NC 27710 USA; ^10^ Department of Medicine Duke University School of Medicine Durham NC 27710 USA; ^11^ Department of Cancer Biology and Colorectal Surgery Lerner Research Institute, Cleveland Clinic Cleveland OH 44195 USA; ^12^ Center for Excellence in Biomacromolecules Chinese Academy of Sciences Beijing 100101 China

**Keywords:** chromatin remodeling, colorectal cancer, DPP4, epigenetics, hepatic growth factor (HGF), metastasis, PU.1

## Abstract

Colorectal cancer (CRC) metastasizes mainly to the liver, which accounts for the majority of CRC‐related deaths. Here it is shown that metastatic cells undergo specific chromatin remodeling in the liver. Hepatic growth factor (HGF) induces phosphorylation of PU.1, a pioneer factor, which in turn binds and opens chromatin regions of downstream effector genes. PU.1 increases histone acetylation at the DPP4 locus. Precise epigenetic silencing by CRISPR/dCas9^KRAB^ or CRISPR/dCas9^HDAC^ revealed that individual PU.1‐remodeled regulatory elements collectively modulate DPP4 expression and liver metastasis growth. Genetic silencing or pharmacological inhibition of each factor along this chromatin remodeling axis strongly suppressed liver metastasis. Therefore, microenvironment‐induced epimutation is an important mechanism for metastatic tumor cells to grow in their new niche. This study presents a potential strategy to target chromatin remodeling in metastatic cancer and the promise of repurposing drugs to treat metastasis.

## Graphical abstract

Metastasis of colorectal cancer to the liver is a leading cause of cancer‐related death. This work shows that metastatic colorectal cancer cells undergo chromatin remodeling in the liver via an HGF‐PU.1‐DPP4 axis. Pharmacological or genetic inhibition of this remodeling axis can suppress liver metastasis and prolong survival.

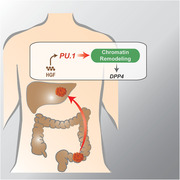

## 1 Introduction

Metastasis of cancer cells from primary sites to distant organs accounts for the majority of cancer‐related deaths. Colorectal cancer (CRC) is a leading cause of cancer mortality in the United States, and its 5‐year survival rate drops significantly once patients develop distant metastases,^[^ ^1^ ^]^ in which liver is the most common site for CRC metastases.^[^ ^2^, ^3^ ^]^

CRC metastases have not been consistently associated with specific oncodriver mutations.^[^ ^4^, ^5^, ^6^, ^7^ ^]^ Therefore, current CRC chemotherapy does not distinguish between different metastatic loci. Nevertheless, metastatic cells have to adapt to their new niche as suggested by the “seed and soil” theory,^[^ ^8^ ^]^ in which microenvironment factors including the extracellular matrix (ECM), vasculature, secreted factors, and myeloid cells can elicit changes in metastatic tumor cells.^[^ ^9^, ^10^ ^]^ Mixed response to chemotherapy between primary CRC tumors and metastases occurs in a significant fraction of patients,^[^ ^11^ ^]^ supporting the existence of important biological differences between the two.

The liver environment is different from that of the colon in terms of cellular composition and metabolism. Metabolic reprogramming has been shown to promote CRC liver metastasis.^[^ ^12^, ^13^, ^14^, ^15^ ^]^ In this study, we applied epigenetic and transcriptomic profiling techniques, including transposase‐accessible chromatin and sequencing (ATAC‐seq), multiplexed indexed T7 chromatin immunoprecipitation (Mint‐ChIP), and RNA‐seq, on synchronous primary CRC tumors and liver metastases from an in vivo CRC orthotopic‐metastasis model, which revealed systematic chromatin alterations between liver metastases and primary tumors. Cross‐validation with clinical datasets identified a major chromatin remodeling axis whereby hepatic growth factor (HGF) induces phosphorylation of PU.1, a pioneer transcription factor that opens up closed chromatin and recruits additional epigenetic modifiers.^[^ ^16^, ^17^, ^18^, ^19^ ^]^ PU.1 binding was enriched in chromatin regions that became accessible in liver metastases. In particular, it activated dipeptidylpeptidase 4 (DPP4) through its three enhancers and promoter, each of which was shown to modulate liver metastasis in vivo by CRISPR/dCas9^KRAB^. Pharmacological inhibition or genetic silencing of HGF, PU.1, or DPP4 suppressed liver metastases.

## 2 Results

### 2.1 Chromatin Remodeling Occurs in CRC Liver Metastases

We used an in vivo orthotopic CRC metastatic model obtained by injecting mCherry‐ and luciferase‐labeled human CRC cells into the cecal wall of immunocompromised NOD.Cg‐*Prkdc^scid^ Il2rg^tm1wjl^/*SzJ (NSG) mice as previously described.^[^ ^12^, ^20^ ^]^ We performed cecum injection with cells from a common CRC line, HT29, and three liver metastases patient‐derived xenografts (PDX) – CRC57, CRC12x, and CRC247 (Table S1, Supporting Information). The injected CRC cells first formed cecal tumors then metastasized to the liver.

To investigate changes in the open chromatin state in primary CRC and liver metastases, we performed ATAC‐seq on the primary CRC and liver metastatic CRC tumors collected from the in vivo model five weeks after injection (Figure S1A, Supporting Information).^[^ ^21^ ^]^ To avoid contamination from stromal cells, harvested CRC tumors from ceca and livers of tumor‐bearing mice were dissociated into single cells and subjected to fluorescence‐activated cell sorting (FACS) based on mCherry fluorescence levels (Figure S1B, Supporting Information).

**Figure 1:**
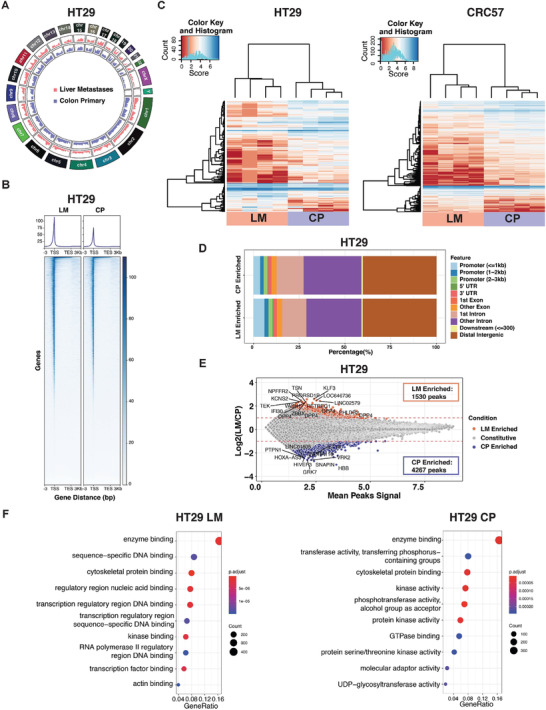
Epigenetic profiling on CRC liver metastases and CRC primary tumor. A) Circos plot revealing global chromatin accessibility change across different chromosomes in HT29 liver metastases versus primary CRC tumor. B) Heatmap for chromatin accessibility across gene body from TSS to transcription end site (TES) with 3 kb flanking regions in HT29 liver metastases versus primary CRC tumor. C) Hierarchical heatmap of 1000 chromatin accessible regions with the highest variance between liver metastases and primary CRC tumor in HT29 and CRC57 across four biological replicates. D) Distribution of enriched chromatin accessible regions across the genome in HT29 liver metastases versus primary CRC tumor. E) MA‐plot showing the differentially enriched (DE) accessible chromatin regions in HT29 liver metastases versus primary CRC tumor. F) Gene Ontology (GO) analysis on molecular functions for DE accessible chromatin regions in HT29 liver metastases versus primary CRC tumor. LM, liver metastases. CP, CRC primary tumor.

We obtained high‐quality ATAC‐seq libraries from both primary and metastatic tumors with consistency across biological replicates (*N* = 4 mice), as reflected by plots of irreproducible discovery rate (IDR) and representative fragment distribution (Figure S1C,D, Supporting Information). Across the four CRC models, the global chromatin accessibility between liver metastases and primary CRC was largely similar, as shown by Circos plots (**Figure** **1**A and Figure S1E, Supporting Information), as was the overall chromatin accessibility around a gene body (Figure 1B and Figure S1F, Supporting Information). These results suggest that metastatic cells mostly retain their CRC identity. Nevertheless, a fraction of the chromatin accessibility peaks was significantly different between primary and metastatic tumor cells according to DiffBind,^[^ ^22^ ^]^ as shown by hierarchical heatmaps of the 1000 open chromatin regions with the highest variance (Figure 1C and Figure S1G, Table S5, Supporting Information). Most of the altered regions were in intronic or intergenic regions where *cis*‐regulatory elements or enhancers locate (Figure 1D and Figure S1H, Supporting Information). We further associated the altered chromatin regions with putative genes under their control by annotating to the nearest transcription start sites (TSS) (Figure 1E and Figure S1I, Supporting Information). Gene Ontology analyses demonstrated the upregulation of transcription regulation and DNA binding pathways in the liver metastases compared to the primary CRC (Figure 1F and Figure S1J, Supporting Information).^[^ ^23^ ^]^ Collectively, the ATAC‐seq analyses suggest that although metastatic cells largely resemble primary CRC cells, they undergo distinct chromatin remodeling.

### 2.2 DPP4 is Upregulated in CRC Liver Metastases

**Figure 2:**
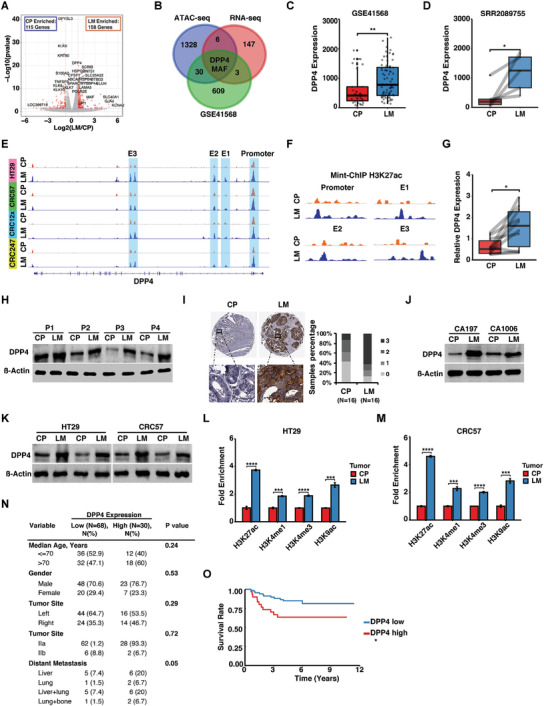
DPP4 is upregulated in CRC liver metastases. A) RNA‐seq volcano‐plot showing DE genes detected in HT29 liver metastases versus primary CRC tumor. B) Integrated analysis of ATAC‐seq, RNA‐seq, and GEO dataset (GSE41568) of upregulated genes in liver metastases. C) Analysis of differential DPP4 expression in GEO dataset (GSE41568) between primary CRC and liver metastases. D) Analysis of differential DPP4 expression in the clinical RNA‐seq dataset SRR2089755 from five matched patient liver metastases versus primary CRC tumor. E) ATAC‐seq signal track showing DPP4‐associated open chromatin in liver metastases versus primary CRC tumor. F) Mint‐ChIP signal track showing H3K27ac histone activation markers in the DPP4 promoter and three enhancers in liver metastases versus primary CRC. G) RT‐qPCR showing DPP4 expression levels in patient liver metastases versus primary CRC tumors. H) Western blots showing DPP4 expression levels in paired primary CRC and liver metastases collected from four patients. I) Representative IHC staining and evaluation of DPP4 expression measured on a tissue microarray that contains 16 paired primary CRC and liver metastases. (Scale bar, 50 um). J) Western blots showing DPP4 expression levels in two paired patient‐derived liver metastases and primary CRC organoids. K) Western blots showing DPP4 expression levels in primary CRC and liver metastases derived from cecum‐injected‐HT29 and ‐CRC57 cells. L,M) ChIP‐qPCR analysis showing changes in H3K27ac, H3K4me1, H3K4me3, and H3K9ac levels on DPP4 promoter in liver metastases versus primary CRC tumor. N) Correlation of DPP4 expression and clinicopathological features of 98 CRC patients. O) Kapla–Meier analysis of relapses of CRC patients with high (*N* = 30) and low (*N* = 68) DPP4 expression levels in the tumors. LM, liver metastases. CP, CRC primary tumor. E1, E2, and E3, enhancer 1, enhancer 2, and enhancer 3. Data represent the mean ± s.e.m. in (C) and (D), and mean ± s.d. in (G), (L), and (M). *p*‐values were calculated based on Student's *t*‐test in (C), (D), (G), (L), (M), and (N), and log‐rank test in (O). *p < 0.05; **p < 0.01; ***p < 0.001; ****p < 0.0001.

We performed RNA‐seq to compare the transcriptomic states between primary CRC and liver metastases collected from the in vivo model. Consistent with the ATAC‐seq results, a fraction of genes was differentially expressed (DE) although most remained unchanged (**Figure** **2**A, Table S6, Supporting Information). Integrated analysis of the ATAC‐seq and RNA‐seq profiling identified eight genes that are enriched in liver metastases (Figure 2B). We cross‐validated the upregulated genes with an RNA microarray dataset that contains 39 primary CRC and 74 liver metastases from stage IV CRC patients (GEO:GSE41568).^[^ ^24^ ^]^ DPP4 and MAF were the most upregulated in the liver metastases in all three datasets (Figure 2B,C, and Figure S2A, Supporting Information). We further validated the expression of DPP4 and MAF in another clinical dataset containing RNA‐seq results from paired patient primary CRC and liver metastases (SRR2089755), and both of the genes were significantly upregulated in liver metastases (Figure 2D and Figure S2B, Supporting Information). In liver metastatic CRC cells, DPP4 had enriched open chromatin in its promoter and three enhancer regions (Figure 2E), but MAF only had open chromatin changes around its promoter (Figure S2C, Supporting Information).

We supplemented the ATAC‐seq open chromatin maps with high‐resolution annotation of the H3K27ac histone activation marker using Mint‐ChIP, a technique that requires far fewer cells than conventional ChIP‐seq and is hence more compatible with the in vivo metastasis model.^[^ ^25^ ^]^ Consistent with chromatin accessibility results, Mint‐ChIP showed that the DPP4 promoter and three enhancers harbored more H3K27ac histone activation markers in CRC liver metastases than in primary CRC tumors (Figure 2F and Figure S2D, Supporting Information).

To further confirm the upregulation of DPP4 in clinical CRC liver metastases, we acquired tissues from ten paired primary CRC and liver metastases (Table S2, Supporting Information). RT‐qPCR showed that DPP4 was upregulated in liver metastases (Figure 2G). The four sample pairs with enough remaining tissue mass for Western blot analysis had higher DPP4 protein levels in liver metastases versus primary CRC (Figure 2H). IHC staining of a tissue array containing paired primary CRC tumors and liver metastases from 16 patients also showed higher DPP4 staining levels in liver metastases (Figure 2I and Table S3, Supporting Information). We then cultured organoids derived from freshly collected specimens of synchronous primary CRC and liver metastases from two patients (Figure S3A and Table S4, Supporting Information). Consistent with our in vivo results, DPP4 protein levels were higher in liver metastases‐derived organoids (Figure 2J).

Liver metastases had significantly higher levels of DPP4 than the primary CRC tumors in HT29 and CRC57 from the in vivo orthotopic‐metastasis model (Figure 2K). Consistent with RNA‐seq and ATAC‐seq profiling results, ChIP‐qPCR confirmed that histone activation markers H3K27ac, H3K4me1, H3K4me3, and H3K9ac in the DPP4 promoter were significantly elevated in CRC liver metastases compared to primary CRC (Figure 2L,M). In the two pairs of matched patient‐derived organoids, H3K27ac and H3K4me3 levels in the DPP4 promoter region were consistently higher in liver metastases‐derived organoids than in matched primary CRC‐derived organoids (Figure S3B,C, Supporting Information).

To investigate whether DPP4 expression is correlated with clinical outcomes, we analyzed DPP4 expression and CRC progression based on the data from 98 CRC patients at Catalan Institute of Oncology.^[^ ^26^ ^]^ The patients were divided into two cohorts, DPP4^high^ and DPP4^low^. 40% of DPP4^high^ patients developed liver metastasis, while only 14.8% DPP4^low^ patients developed liver metastasis (Figure 2N). In addition, DPP4^high^ patients had significantly higher rates of relapse and metastasis than the DPP4^low^ patients (Figure 2O). Together, these data suggested that chromatin remodeling upregulates DPP4 in CRC liver metastases, which is associated with poor prognosis.

### 2.3 Silencing of DPP4 Suppresses CRC Liver Metastases

**Figure 3:**
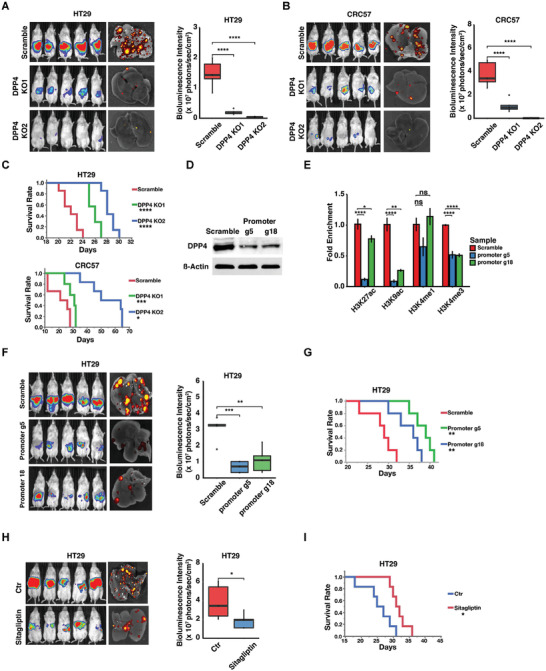
Ablation of DPP4 suppresses CRC progression. A,B) Images and quantifications of bioluminescence of NSG mice injected with A) luciferase‐labeled HT29 and B) CRC57 cells carrying scrambled (control) or DPP4 knockout (DPP4 KO1 or KO2) gRNA constructs. C) Survival analysis of NSG mice injected with luciferase‐labeled HT29 and CRC57 cells carrying scrambled (control) or DPP4 knockout (DPP4 KO1 or KO2) gRNA constructs. D) Western blots showing downregulation of DPP4 by selected and scrambled gRNA tested in the CRISPR/dCas9^HDAC^ system. E) ChIP‐qPCR showing the effect of CRISPR/dCas9^HDAC^ system on DPP4 promoter‐associated histone modification. F,G) Images and quantifications of F) bioluminescence and G) survival analysis of NSG mice carrying scrambled and selected gRNA in the CRISPR/dCas9^HDAC^ system. H,I) Images and quantifications of H) bioluminescence and I) survival analysis of NSG mice receiving spleen‐injection of HT29 cells with or without Sitagliptin oral gavage. Data represent the mean ± s.d. *p*‐value was calculated based on student's *t*‐test in (H), ANOVA and Tukey's HSD post doc test in (A), (B), (E), and (F), and log‐rank test in (C), (G), and (I). *p < 0.05; **p < 0.01; ***p < 0.001; ****p < 0.0001.

To investigate how DPP4 regulates tumor development, we used CRISPR/Cas9 with two independent guide RNAs (gRNAs) to knock out DPP4 in HT29 and two of the PDX cell lines, CRC57 and CRC247. DPP4 knockout efficiency was validated by Western blot (Figure S4A,B,C, Supporting Information). Orthotopic injection mouse model showed that DPP4 knockout suppressed liver metastases and prolonged survival (**Figure** **3**A–C, Figure S4D,E, Supporting Information). We further knocked out DPP4 using CRISPR/Cas9 in patient‐derived CRC organoids (Figure S4F, Supporting Information) and intrahepatically injected them into mice. Silencing of DPP4 significantly suppressed tumor growth in the liver compared to the control (Figure S4G, Supporting Information). We then knocked down *Dpp4* in mouse CRC CT26 cells and injected them into the ceca of syngeneic immunocompetent BALB/c mice (Figure S4H, Supporting Information). shRNA, rather than CRISPR/Cas9, was used to silence *Dpp4* to avoid potential complications by the host immune response to CRISPR/Cas9 constructs.^[^ ^27^, ^28^ ^]^ *Dpp4* knockdown suppressed CT26 liver metastases in the immunocompetent mice and prolonged their survival (Figure S4I,J, Supporting Information).

### 2.4 Epigenetic Silencing of DPP4 Regulatory Elements Suppresses CRC Liver Metastases

To unravel which DPP4 regulatory elements contribute to liver metastasis, we used CRISPR/dCas9^KRAB^ system to precisely silence each of the three enhancers and promoter.^[^ ^29^, ^30^ ^]^ We designed 15, 18, 16, and 29 gRNAs spanning the entire regions of the three enhancers and promoter, respectively (Figure S5A, Supporting Information), and screened for DPP4 downregulation by RT‐qPCR (Figure S5B, Supporting Information). The gRNAs targeting each regulatory element with the highest downregulation efficiency on DPP4 were selected and validated by Western blot (Figure S5C, Supporting Information). We then injected HT29 cells carrying CRISPR/dCas9^KRAB^ and the selected or scrambled gRNA into the mouse ceca. Each selected gRNA reduced tumor growth and prolonged survival compared to the scrambled control (Figure S5D,E, Supporting Information). Therefore, in addition to the promoter, each of the three DPP4 enhancers located in the opened chromatin modulates CRC liver metastases.

We then used CRISPR/dCas9^HDAC^ to manipulate histone acetylation within the DPP4 promoter.^[^ ^31^ ^]^ We screened six DPP4 gRNAs targeting the opened chromatin regions in the DPP4 promoter (Figure S5F, Supporting Information) and the two selected (g5 and g18) significantly suppressed H3K27ac, H3K9ac and H3K4me3 levels (Figure 3D,E). We then orthotopically injected the CRC cells carrying the scrambled or selected gRNAs into the mouse ceca. The selected gRNAs suppressed tumor growth and metastases and prolonged overall survival (Figure 3F,G).

### 2.5 A Pharmacological DPP4 Inhibitor Suppresses CRC Liver Growth

Sitagliptin is a DPP4 inhibitor that has been approved for clinical treatment of type II diabetes.^[^ ^32^ ^]^ We investigated whether Sitagliptin could prolong survival of metastatic liver disease by using a spleen‐injection liver metastasis model, in which injected CRC cells reach the liver to form metastases without first forming primary tumors, so that liver metastases are the sole contributor to death.^[^ ^33^ ^]^ Sitagliptin treatment, which started three days after spleen injection, significantly suppressed CRC growth in the liver (Figure 3H) and increased the survival of tumor‐bearing mice (Figure 3I).

### 2.6 DPP4 Suppresses Tumor‐Killing Neutrophils

Notably, downregulation of DPP4 did not affect migration and proliferation of CRC cells in vitro (Figure S6, Supporting Information). DPP4 has been shown to truncate the neutrophil chemoattractants CXCL6 and CXCL10.^[^ ^34^, ^35^, ^36^, ^37^ ^]^ NSG mice lack mature T cells, B cells, and NK cells and have defective dendritic cells and macrophages, but their neutrophils are intact.^[^ ^38^ ^]^ We analyzed neutrophil accumulation in the primary CRC and liver metastases in orthopotic injection mouse model, finding that knocking out DPP4 in CRC cells significantly increased neutrophil accumulation in both primary CRC and liver metastases (Figure S7A–C, Supporting Information). Consistently, the intrahepatipic injection model also showed that knocking out DPP4 increased neutrophil cell infiltration in the liver (Figure S7D,E, Supporting Information). We further analyzed the correlation between DPP4 and Myeloperoxidase (neutrophil marker gene) expression in clinical CRC liver metastases by integrating multiple GEO datasets (GSE40367, GSE41258, and GSE49355). The neutrophil content is inversely correlated with DPP4 expression in clinical CRC liver metastases (Figure S7F, Supporting Information). Independently, immunochemistry on a CRC tissue array containing 16 paired primary CRC tumors and liver metastases indicated that DPP4 expression is much higher in liver metastases than that in primary tumors (Figure 2I). Conversely, neutrophil content is low in liver metastases and high in primary tumors (Figure S7G, Supporting Information).

Tumor‐associated neutrophils have been shown to kill tumor cells by physical contact.^[^ ^39^ ^]^ We isolated and co‐cultured neutrophils and DPP4 knockout CRC cells from the same HT29 or CRC57 tumors. Time‐lapse microscopy showed that DPP4 knockout CRC cells had enhanced neutrophil recruitment for physical contact (Figure S7H and Movies S1,S2, Supporting Information). We then performed a trans‐well migration assay with active CXCL6 or DPP4‐truncated CXCL6 in the lower chamber and neutrophils isolated from the tumor‐bearing mouse in the top chamber. Both human and mouse CXCL6 significantly enhanced neutrophil migration compared to the truncated CXCL6 (Figure S7I, Supporting Information). To further evaluate the cytotoxic effect of CRC‐associated neutrophils, we isolated neutrophils from tumor‐bearing mice and co‐cultured with luciferase‐labeled HT29 and CRC57 cells. DPP4 knockout enhanced killing of tumor cells by neutrophils compared to the scrambled control (Figure S7J, Supporting Information).

### 2.7 Pioneer Factor PU.1 Regulates DPP4 via its Enhancers and Promoter

**Figure 4:**
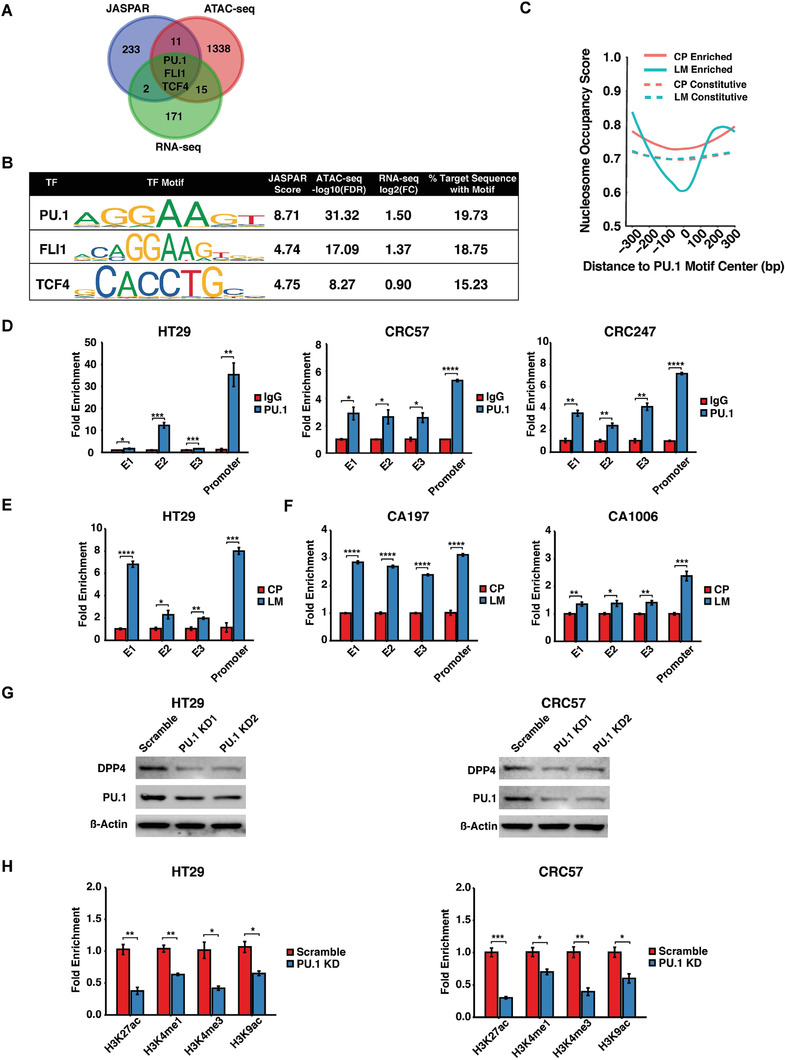
PU.1 upregulates DPP4 expression through remodeling DPP4‐associated chromatin. A) Integrated analysis of JASPAR, RNA‐seq, and ATAC‐seq revealing three potential DPP4 epigenetic regulators. B) Corresponding scores of the potential DPP4 regulators. C) Nucleosome occupancy analysis of enriched or constitutive peaks from liver metastases or primary CRC centered at PU.1 motif with 300 bp flanking window. D) ChIP‐qPCR showing PU.1 binding in DPP4 promoter and enhancer regions. E,F) ChIP‐qPCR showing PU.1 binding to DPP4 promoter and enhancers in HT29‐derived liver metastases versus primary tumors from E) CRC orthotopic‐model, and F) patient‐derived CRC organoids. G) Western blots showing DPP4 expression levels in HT29 or CRC57 carrying scrambled (control) or PU.1 knockdown (PU.1 KD1 or KD2) shRNAs constructs. H) ChIP‐qPCR showing relative H3K27ac, H3K4me1, H3K4me3, and H3K9ac enrichments in HT29 or CRC57 carrying scrambled (control) or PU.1 knockdown (PU.1 KD1) shRNA constructs. LM, liver metastases. CP, CRC primary tumor. E1, E2, and E3, enhancer 1, enhancer 2, and enhancer 3. Data represent the mean ± s.d. *p*‐values were calculated based on Student's *t*‐test. *p < 0.05; **p < 0.01; ***p < 0.001; ****p < 0.0001.

We analyzed binding motifs of potential upstream transcription factors using JASPAR.^[^ ^40^ ^]^ Among the 249 predicted transcription factors, 14 had enriched chromatin accessible regions in liver metastases; upon RNA‐seq analysis, PU.1, FLI1, and TCF4 were ranked as the top three (**Figure** **4**A). Among these, PU.1 had the best scores in all categories (Figure 4B).

PU.1 is a pioneer factor that opens up closed chromatin and recruits additional epigenetic modifiers to regulate hematopoiesis and fibrosis.^[^ ^18^, ^19^ ^]^ Nucleosome occupancy analysis by NucleoATAC^[^ ^41^ ^]^ demonstrated that nucleosomes are depleted around the putative PU.1 binding motifs in liver metastasis‐enriched open chromatin regions; conversely, there is little nucleosome remodeling in the primary CRC cells or in regions constitutively open in both primary CRC and liver metastases (Figure 4C). ChIP‐qPCR confirmed PU.1 binding to the DPP4 promoter and enhancer regions in HT29, CRC57, and CRC247 cells (Figure 4D). Moreover, PU.1 binding to the DPP4 promoter and three enhancer regions was significantly enriched in CRC cells isolated from the liver metastases relative to cells isolated from the primary CRC tumors (Figure 4E). ChIP‐qPCR in the aforementioned organoids derived from synchronous patient primary CRC and liver metastatic tumors (Figure S3A, Table S4, Supporting Information) further confirmed the enrichment of PU.1 binding in liver metastases (Figure 4F). PU.1 knockdown suppressed DPP4 expression (Figure 4G) and decreased H3K27ac, H3K4me1, H3K4me3, and H3K9ac histone markers in DPP4 promoter‐associated chromatin regions (Figure 4H).

### 2.8 PU.1 Promotes CRC Liver Metastasis

**Figure 5:**
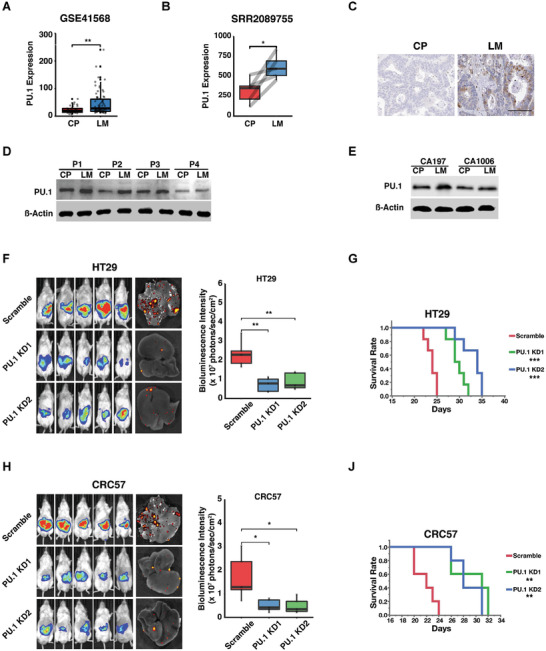
PU.1 is upregulated in CRC liver metastases and promotes CRC progression. A) Analysis of differential PU.1 expression in GEO dataset (GSE41568) between primary CRC and liver metastases. B) Analysis of differential PU.1 expression in the RNA‐seq dataset SRR2089755 from five matched patient primary CRC and liver metastases. C) IHC staining of differential PU.1 expression between primary CRC and liver metastases. (Scale bar, 100 um). D) Western blots showing DPP4 expression levels in paired primary CRC and liver metastases collected from four patients. E) Western blots showing DPP4 expression levels in paired primary CRC and liver metastases‐derived organoids collected from two patients (CA197 and CA1006). F,G) Images and quantification of F) bioluminescence and G) survival analysis of NSG mice injected with luciferase‐labeled HT29 carrying scrambled (control) or PU.1 knockdown (PU.1 KD1 or KD2) shRNA constructs. H,J) Images and quantification of H) bioluminescence and J) survival analysis of NSG mice injected with luciferase‐labeled CRC57 carrying scrambled (control) or PU.1 knockdown (PU.1 KD1 or KD2) shRNA constructs. LM, liver metastases. CP, CRC primary tumor. Data represent the mean ± s.e.m. in (A) and (B), and the mean ± s.d. in (F) and (H). *p*‐values were calculated based on Student's *t*‐test (A) and (B), log‐rank test in (G) and (J), and ANOVA and Tukey's HSD post hoc test in (F) and (H). *p < 0.05; **p < 0.01; ***p < 0.001; ****p < 0.0001.

Consistent with the results from RNA‐seq profiling of the in vivo model, GSE41568 and SRR2089755 analysis, and IHC staining showed elevated PU.1 expression in clinical CRC liver metastases compared to primary CRC (**Figure** **5**A–C), which was further validated by Western blot of aforementioned pairs of primary CRC and liver metastases tissue samples and patient‐derived organoids (Table S2,S4, Supporting Information) (Figure 5D,E). PU.1 knockdown reduces the sizes of liver metastases (Figure 5F,H) and prolonged survival (Figure 5G–J) of mice bearing either HT29 or CRC57 tumors.

### 2.9 HGF Upregulates DPP4 through Activation of PU.1

**Figure 6:**
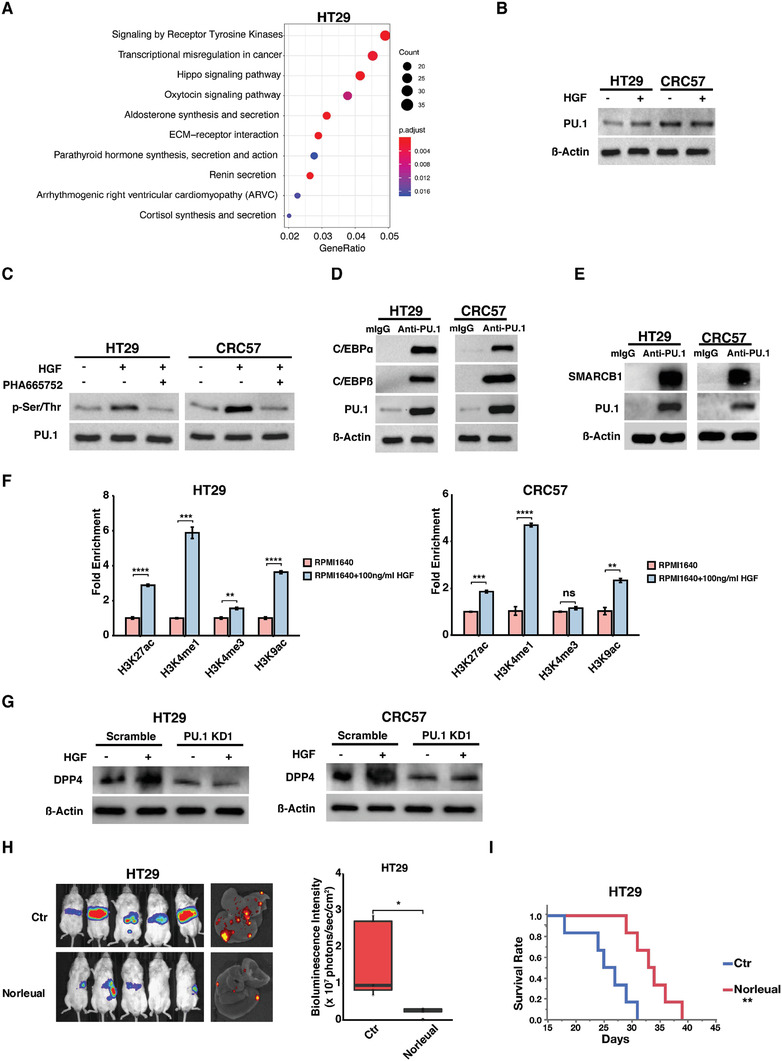
HGF upregulates DPP4 through activaton of PU.1. A) Dotplot of pathway analysis on enriched accessible chromatin regions in HT29 liver metatases. B) Western blots showing PU.1 expression level in HT29 or CRC57 cells treated with or without HGF. C) Western blots showing PU.1 phosphorylation in HT29 or CRC57 cells treated with or without HGF or the c‐Met inhibitor PHA665752. D,E) Western blots showing that PU.1 complexed with D) C/EBP*α* and ‐*β* and E) SMARCB1. F) ChIP‐qPCR showing relative H3K27ac, H3K4me1, H3K4me3, and H3K9ac enrichments in HT29 or CRC57 cultured in RPMI1640 media supplemented with or without 100 ng mL^−1^ HGF. G) Western blots showing the expressions of DPP4 in HT29 or CRC57 carrying scrambled (control) or PU.1 knockdown shRNA cultured in RPMI media supplemented with or without 100 ng mL^−1^ HGF. H,I) Images and quantification of H) bioluminescence and I) survival analysis of NSG mice spleen‐injected with luciferase‐labeled HT29 cells receiving i.p. administered PBS (control) or the HGF inhibitor Norleual. Data represent the mean ± s.d. in (F) and (H). *p*‐values were calculated based on Student's *t*‐test (F) and log‐rank test in (I). *p < 0.05; **p < 0.01; ***p < 0.001; ****p < 0.0001.

PU.1 activation requires phosphorylation to enhance its DNA binding and co‐factor recruitment.^[^ ^42^ ^]^ Pathway analysis based on liver metastases‐enriched open chromatin regions identified by ATAC‐seq showed that receptor tyrosine kinase (RTK) pathways were consistently activated in liver metastases of mice injected with each of the four tested CRC cell lines (**Figure** **6**A and Figure S8, Supporting Information). HGF is highly expressed in the liver microenvironment and can facilitate tumor growth.^[^ ^43^ ^]^ Moreover, the HGF/c‐Met pathway is one of the most important RTK pathways in both the liver and advanced hepatocellular carcinoma.^[^ ^44^ ^]^ Recombinant HGF did not affect PU.1 expression (Figure 6B) but significantly enhanced PU.1 phosphorylation in HT29 and CRC57 cells, which was abrogated by the c‐Met inhibitor PHA665752 (Figure 6C).

Phosphorylated PU.1 has been reported to initiate chromatin remodeling through complexing with C/EBP*α*/*β* and SWI/SNF‐related matrix‐associated actin‐dependent regulator of chromatin subfamily B member 1 (SMARCB1).^[^ ^45^ ^]^ Immunoprecipitation confirmed that PU.1 complexed with C/EBP*α*/*β* and SMARCB1 in HT29 and CRC57 cells (Figure 6D,E). HGF treatment significantly increased the levels of H3K27ac, H3K4me1, and H3K9ac markers in the DPP4 promoter region (Figure 6F). Recombinant HGF upregulated DPP4 expression and this was abrogated by PU.1 knockdown, which suggests that HGF enhances DPP4 expression via PU.1 (Figure 6G).

We then evaluated the therapeutic potential of Norleual, an HGF inhibitor, on prolonging survival of metastatic liver disease using the spleen injection liver metastasis model. Intraperitoneal (i.p.) injection of Norleual, which started three days after spleen injection, reduced tumor sizes in the liver and prolonged survival (Figure 6H,I).

## 3 Discussion

Although CRC liver metastases are not associated with any unique oncodriver mutation, this study demonstrates that metastatic cells growing in the liver have undergone chromatin remodeling. We identified a chromatin remodeling axis whereby HGF in the liver environment induces phosphorylation of the pioneer factor PU.1 in metastatic cells. Phosphorylated PU.1 modulates downstream regulatory elements to activate effector genes such as DPP4, which promotes tumor growth. Targeting the factors or regulatory elements along the axis resulted in strong anti‐tumor effects, which suggests that targeting this axis may be a viable strategy against liver metastasis. For instance, DPP4 expression has been associated with poor CRC prognosis,^[^ ^46^ ^]^ and a recent epidemiology study revealed that diabetic patients with CRC who were treated with DPP4 inhibitors had a statistically significant survival advantage.^[^ ^47^ ^]^ The HGF/PU.1/DPP4 axis is unlikely the only major chromatin remodeling axis in CRC liver metastasis, as PU.1 have other downstream targets while the DPP4 enhancers have other upstream regulators. More generally, since metastatic cancers of various types have not been consistently associated with specific driver mutations,^[^ ^7^ ^]^ targeting chromatin remodeling pathways of metastatic cancers may provide additional treatment options in the future.

Previous reports showed that inhibition of DPP4 induced breast and prostate cancer metastasis via the CXCL12/CXCR4/mTOR axis and epithelial‐to‐mesenchymal transition (EMT).^[^ ^48^, ^49^ ^]^ This highlights the importance of context, which includes cancer type, stage, and microenvironment. For example, Notch receptor can act both as oncogene or tumor suppressor in different cancer types.^[^ ^50^ ^]^ TGF‐*β* functions as tumor suppressor in early‐stage CRC but promotes metastasis and immune evasion in late stage and metastases.^[^ ^51^, ^52^ ^]^ Similarly, DPP4 seems to play dual roles in different cancer types and highlights the opposing forces between cancer cell intrinsic mechanisms and the microenvironment.

Unlike the colon, the liver environment consists of hepatocytes, Kupffer cells, stellate cells, and sinusoidal endothelial cells (LSECs).^[^ ^53^ ^]^ Several major hepatic acellular factors, including HGF, CXCL8, VEGF, MAPK, TGF‐*β*, NF‐*κ*B, and CEA, have been shown to promote liver metastasis via the promotion of proliferation, angiogenesis, EMT, and cell attachment.^[^ ^54^, ^55^, ^56^, ^57^, ^58^, ^59^, ^60^ ^]^ In addition to these hepatic factors, hormones, and cytokines, such as insulin, IGF‐1, estrogen, and bile acid, make the liver a unique environment.^[^ ^61^ ^]^ The development of a metastatic niche following the arrival of tumor cells is critical to liver metastases and progression.^[^ ^62^ ^]^ For instance, the IL‐6–STAT3–SAA pathway in hepatocytes establishes a pro‐metastatic niche for the seeding and growth of pancreatic cancer in the liver.^[^ ^63^ ^]^ Therefore, HGF is unlikely the sole factor; rather, it is probably among a group of hepatic cytokines, chemokines, hormones, and growth factors that drives reprogramming of CRC cells.

The “seed and soil” hypothesis proposed that interactions between metastatic cells and their organ microenvironment are important and may play a role in organ tropism.^[^ ^8^ ^]^ As metastatic loci have not been consistently associated with specific oncodriver mutations,^[^ ^4^, ^5^, ^6^, ^7^ ^]^ our study suggests that epimutations may provide an alternative mechanism for metastatic cells to adapt to and evolve in the new niche. However, like genetic mutations, most epimutations are likely “passengers” that do not have any functional consequence. By combining footprinting analysis, nucleosome occupancy analysis, and CRISPR/dCas9^KRAB^ or CIRSPR/dCas9^HDAC^ editing, we managed to identify and validate chromatin‐remodeling transcription factors and regulatory elements that functionally impact metastatic tumors. Identification of these oncodriver epimutations may explain the lack of metastasis‐specific oncodriver mutations and hold the key to understanding interactions between metastatic tumor cells and their niche for new therapeutic development.

## 4 Experimental Section

### Animal Procedures

All animal procedures were approved by Duke University DLAR following the protocol A286‐15‐10. NOD.*Cg‐Prkdc^scid^il2rg^tm1Wjl^*/SzJ (NSG) mice and BALB/c mice used in this study were 8 weeks old. To test DPP4 inhibition in vivo, three days after spleen injection, Sitagliptin (Millipore Sigma, Cat#Y0001812) was administered to mice twice a day via oral gavage at 250 mg kg^−1^.^[^ ^64^ ^]^ To test HGF inhibition in vivo, three days post spleen injection, Norleual (Tocris, Cat#5369) was administered to mice i.p. twice a day at 1 mg kg^−1^.^[^ ^65^ ^]^

### Cell Culture

Human CRC cell line HT29, patient derived xenograft (PDX) human CRC cell lines CRC57, CRC247, and CRC12x, and BALB/c mouse syngeneic colon cancer cell line CT26 were used in the study. These cell lines were cultured in RPMI 1640 complete medium supplemented with 10% FBS and 1% penicillin streptomycin solution. Engineered cell lines expressing mCherry and luciferase were established according to the published procedure.^[^ ^12^ ^]^

### Western Blot Analysis

Western blot analysis was performed following the previously described protocol.^[^ ^66^ ^]^ Antibodies used in this study include anti‐human DPP4 antibody (1:1000, Abcam, ab28340), anti‐human PU.1 antibody (1:1000, Abcam, ab88028), anti‐phospho‐(Ser/Thr) Phe antibody (1:1000, Cell Signaling Technology, 9631S), and anti‐actin (1:1000, Cell Signaling Technology, 4970). Full scans of western blots are included in Figures S9,S10, Supporting Information.

### Cecum Injection, Spleen Injection, Intrahepatic Injection and IVIS Imaging

Cecum injection and spleen injection were performed following the previously published procedures.^[^ ^67^, ^68^, ^69^ ^]^ Briefly, 1 × 10^6^, 1 × 10^5^, or 2.5 × 10^5^ engineered cells carrying luciferase/mCherry were injected into the mouse cecal wall, spleen, or liver, respectively. Spleen removal was performed following the described protocol.^[^ ^33^ ^]^ The IVIS luciferase imaging system 200 (Xenogen) was used to detect the luciferin luminescent signal to examine in vivo tumor development and liver metastases after sacrificing the mice.

### ATAC‐seq and RNA‐seq

Four weeks post cecum injection, primary cecum tumors and metastatic liver tumors were dissociated into single cells using the human tumor dissociation kit (Miltenyi Biotec, Cat#130‐095‐929). CRC cells were sorted by FACS (SONY SH800) based on mCherry signal. ATAC‐seq libraries were made from 50 000 CRC cells of each biological replicate following the previously described protocol,^[^ ^21^ ^]^ and the tagmented nuclei was sequenced using an Illumina PE, 2 × 150 bp. Total RNA was isolated using Qiagen RNeasy Mini kit according to the manufacturer's instructions. RNA‐seq libraries were made and sequenced by Duke GCB by Illumina Hiseq 4000 SE 50 bp.

### TaqMan qPCR

Total RNA was extracted from the cells using Qiagen RNeasy Mini kit. cDNA was synthesized using the High‐Capacity cDNA Archive Kit (Thermofisher Scientific). Quantitative PCR was carried out using the TaqMan Gene Expression Assay (Thermofisher Scientific) to detect DPP4 (Hs00897391).

### Co‐Immunoprecipitation

Immunoprecipitation assays were performed using an immunoprecipitation kit (Abcam, ab206996) and following the manufacturer's instructions. Briefly, the cells were lysed in RIPA buffer, and anti‐human PU.1 antibody (1:100, Thermofisher Scientific, PA5‐17505) was added into the lysate before overnight incubation at 4 °C. Protein G‐conjugated beads were added to pull down the specific protein complex interacting with PU.1. Western blot analysis was performed on the eluted complex using anti‐human C/EBP*α*/*β* (1:1000, Abcam, ab40764, and ab32358) and anti‐human SMARCB1 (1:1000, Thermofisher Scientific, PA5‐40834).

### Chromatin Immunoprecipitation and qPCR

Chromatin immunoprecipitation (ChIP) was performed using the ChromaFlash High‐Sensitivity ChIP Kit (EpiGentek, P‐2027) according to the manufacturer's instructions. Briefly, the cells were fixed by 1% formaldehyde for 10 min at room temperature to crosslink the protein and DNA. Lysis buffer was added, and the cells were centrifuged to collect the chromatin pellets, which were sonicated to generate DNA fragments fewer than 500 base pairs in length and then incubated with the specific antibody overnight at 4 °C. The antibodies in this study included anti‐PU.1 (1:100, Thermofisher Scientific, PA5‐17505), anti‐H3K4me1 (1:100, Abcam, ab8895), anti‐H3K4me3 (1:100, Abcam, ab8580), anti‐H3K9ac (1:100, EpiGentek, A‐4022‐050), and anti‐H3K27ac (1:100, Active motif, 39133). The enriched DPP4 DNA was pulled down by antibody, and qPCR was performed to quantitate DPP4 enrichment. The primers used for qPCR of DPP4‐F and DPP4‐R were AGACTGGCACAGTTTTCTGAG and CTTTCCCATCACCCTTGCTGT, respectively.

### Flow Cytometry for Neutrophil Analysis

Cecum primary tumors and metastatic liver tumors harvested from the cecum injection mouse model were disassociated into single cells using the human tumor dissociation kit (Miltenyi Biotec, Cat#130‐095‐929). Single cells were then incubated with anti‐CD11b‐FITC (1:500, BD Bioscience, 557396) and anti‐Ly6G‐PE (1:500, Thermofisher Scientific, 50‐112‐2445) antibodies. The stained cells were analyzed by flow cytometry.

### Neutrophil Cytotoxic Assay

The neutrophil cytotoxic assay was performed following the protocol described previously.^[^ ^39^ ^]^ Neutrophils were isolated using the MojoSort Mouse Neutrophil Isolation Kit (BioLegend, 480057) from the mouse peripheral blood collected by cardiac punch. Five thousand tumor cells carrying luciferase were seeded into the 96‐well plate four hours before coculture. Then, isolated neutrophils were co‐cultured with tumor cells (100:1) for 12 h. At endpoint, the live cells were measured by the Luminescent Cell Viability Assay (Promega, G7570). During the coculture, live imaging was taken by the ImageXpress Pico System to monitor the process of neutrophil migration.

### Neutrophil Chemotaxis Assay

The neutrophil chemotaxis assay was designed as previously described.^[^ ^70^ ^]^ Briefly, 5 × 10^5^ isolated neutrophils suspended in OptiMEM (Invitrogen) supplemented with 0.5% FBS were seeded into the upper chambers of CytoSelect 96‐Well Transwells (3 µm pore membrane) (Cell Biolabs, CBA‐104/02963‐726). Then, 400 ng human CXCL6 (PeproTech, 300–41), mouse *Cxcl6* (PeproTech, 250‐17), cleaved human CXCL6 and mouse *Cxcl6* by active enzymatic DPP4 (BioLegend, 764102) were added into the lower chamber containing OptiMEM (Invitrogen) supplemented with 0.5% FBS. The transwells were incubated for 5 h at 37 °C and 5% CO2. The migrated neutrophils were measured following the manufacturer's protocol (Cell Biolabs, CBA‐104/02963‐726). The DPP4 activity assay was modified according to the manufacturer's protocol (BioLegend, 764102) and previously described methods.^[^ ^37^ ^]^ For this study, 400 ng DPP4 was incubated with 400 ng hCXCL6 or m*Cxcl6* for 30 min at 37 °C in reaction buffer (25mm Tris‐Cl, PH8.0). After the reaction, DPP4 was removed by 30KWM ultra centrifugal filters (Sigma Millipore, UFC5030).

### Analysis of Tissue Microarrays

Tissue microarrays ZL‐HLin‐Age075Met‐01 (Zhuoli Biotechnology Co, China) were stained using anti‐DPP4 antibody (1:200, Abcam, ab215711) or anti‐Myeloperoxidase (1:100, Abcam, ab208670) and reviewed by a board‐certified pathologist specializing in tumor biology who had no prior knowledge of the patient information for the tumor tissues. Each tissue core on the tissue microarray was given a score of 0–3 based on intensity of staining. Scores of 0 are interpreted as negative for protein expression, and scores of 1, 2, and 3 are interpreted as positive staining for each tissue core.

### Patient Specimens

The primary and liver metastatic CRC specimens were obtained from the 7th Medical Center of PLA General Hospital with informed consent by all donors. All studies were approved by the Ethics Committee of the 7th Medical Center of PLA General Hospital and the Institute of Biophysics, Chinese Academy of Sciences (2020‐39). Matched patient primary tumor and liver organoids were provided under IRB 13–1159 (Cleveland Clinic Foundation). Organoids were screened for mycoplasma contamination and syngeneic identity was confirmed using Short Tandem Repeat (Duke University).

### CRISPR/dCas9^KRAB^ and CRISPR/dCas9^HDAC^ Libarary Design and Screening

To test the potential epigenetic regulatory functions of the four DPP4 regulatory elements (three enhancers and the promoter), we used the CRISPOR algorithm to identify gRNA protospacers within each regulatory region and identify possible off‐targets on other regions of the genome.^[^ ^71^ ^]^ For each regulatory region, the gRNAs were selected to minimize the off‐target alignments. We selected 15, 18, 16, and 29 gRNAs to target enhancer 1, 2, 3, and the promoter of DPP4 to completely span these regions. The gRNAs were put into pLV hU6‐sgRNA hUbC‐dCas9‐KRAB‐T2a‐Puro construct (Addgene, 71236), and were delivered to HT29 cells via lentiviral infection as previously described.^[^ ^30^ ^]^ We followed the previously published method to construct the CRISPR/dCas9^HDAC^ libarary.^[^ ^31^ ^]^ Briefly, the gRNAs targeting DPP4 promoter were cloned into pLV U6‐gRNA‐UbC‐eGFP‐P2A‐Bsr (Addgene 83925), which were delivered to HT29 cells via lentiviral infection together with dCas9‐hHDAC3 construct (Addgene 98591). The sequences of the guide RNAs were shown in Table S7, Supporting Information.

### Quantification and Statistical Anlaysis—ATAC‐seq and RNA‐seq

ATAC‐seq data was processed using the pipeline developed for ENCODE to perform quality control, filtering of low‐quality reads and PCR duplicates, analysis of reproducibility, reference genome alignment, peaks calling, and fold‐enrichment or *p*‐value signal tracks generation. Reads were aligned to reference genome hg19 using Bowtie2. Duplicate and mitochondrial reads were removed, and peaks were called by MACS2.

RNA‐seq raw sequencing reads were quality checked using Fastp^[^ ^72^ ^]^ and summarized with MultiQC.^[^ ^73^ ^]^ Transcripts were aligned to reference genome hg19 using Hisat2^[^ ^74^ ^]^ and quantified by HTSeq.^[^ ^75^ ^]^ EdgeR was used for differential expression analyses.^[^ ^76^ ^]^ Genes DE in liver metastases were classified by *p*‐value ≤ 0.05 and log2FC ≥ 0.8, whereas genes DE in colon primary tumor were classified by *p*‐value ≤ 0.05 and log2FC ≤ −0.8.

### Differential Peak Calling and Annotation

Differential peaks were identified using DiffBind with a minimum of two biological replicates.^[^ ^22^ ^]^ The fold‐changes of peaks were evaluated by the EDGER_BLOCK method in the DiffBind package. Peaks with *p*‐value ≤ 0.05 and |log2FC| ≥ 0.9 were considered “differentially accessible”, where peaks with positive log2FC were enriched in liver metastases and peaks with negative log2FC were enriched in CRC primary tumors. Peaks annotation was performed by using the findMotifsGenome.pl command in the HOMER2^[^ ^77^ ^]^ software based on the nearest TSS. The R package ChIPseeker was also used to annotate peaks and visualization.^[^ ^23^ ^]^ Gene Ontology was performed by the R package clusterProfiler.^[^ ^78^ ^]^

### Nucleosome Occupancy Analysis

For nucleosome occupancy analysis, Broadpeak files per condition were merged and extended by 100 bp on each side. Filtered Bam files were pooled and used as inputs for the NucleoATAC software to obtain occupancy scores.

### Statistical Analysis

Data was expressed as mean ± standard deviation (s.d.) of no smaller than three biological replicates. Student's *t*‐tests were used for comparisons, while ANOVA followed by Tukey's HSD post hoc test (JMP 15) was used for multiple comparisons. All statistical tests were conducted at the two‐sided 0.05 *p*‐value of significance. Patient data were expressed as mean ± standard error of the mean (s.e.m.). Mice were randomly allocated to experimental groups. Survival curves were estimated using the Kaplan–Meier method and compared using the log‐rank test.

## Conflict of Interest

The authors declare no conflict of interest.

## Author contributions

L.W. and E.W. contributed equally to this work. L.W., E.W., P.B., and X.S. came up with the concept, designed the experiments, and wrote the manuscript. E.W. performed the data analysis with help from A.D., K.‐Y.C., and T.C. L.W. performed most experiments with assistance from E.W., J.P.B., Y.W., K.X., Q.H., M.N., W.L., and Y.F. Z.W. performed IHC staining on the tissue array, G.S. analyzed the results from the tissue array staining and L.Z. performed RT‐qPCR and Western blot analysis on the patient samples. Y.K. and P.R.T. helped with the Mint‐ChIP experiment. C.G. helped with CRISPR/dCas9^KRAB^ gRNA design and experimentation. G.C. help with ATAC‐seq protocol optimization and data analysis. V.M. helped with the Kaplan–Meier relapse analysis. E.H. provided the patient organoids in this study. D.H. created the CRC PDX cell lines. J.P.S. and S.K. collected and provided synchronous tissue samples.

## Supporting information

Supporting Information

Supplemental Table 1‐4

Supplemental Table 5

Supplemental Table 6

Supplemental Table 7

Supplemental Movie 1

Supplemental Movie 2

## Data Availability

The data that support the findings of this study are openly available in GEO at reference number GSE153016.
