# Supplementary material for: Chromatin Remodeling of Colorectal Cancer Liver Metastasis is Mediated by an HGF‐PU.1‐DPP4 Axis
Source: Adv Sci (Weinh). 2021 Aug 10;8(19):2004673. doi: 10.1002/advs.202004673 (PMC8498885; doi:10.1002/advs.202004673)
Supplement: Supplementary file 1 — Supporting Information [file ADVS-8-2004673-s003.pdf]

© 2021 Wiley-VCH GmbH

ADVANCED SCIENCE
Open Access

Supporting Information

for *Adv. Sci.*, DOI: 10.1002/advs.202004673

Chromatin Remodeling of Colorectal Cancer Liver Metastasis is Mediated by an HGF-PU.1-DPP4 Axis

Lihua Wang[1,#], Ergang Wang[1,#], Jorge Prado Balcazar[1], Zhenzhen Wu[2,3], Kun Xiang[1], Yi Wang[1], Qiang Huang[1], Marcos Negrete[1], Kai-Yuan Chen[1], Wei Li[1], Yujie Fu[1], Anders Dohlman[1], Robert Mines[1], Liwen Zhang[2,3], Yoshihiko Kobayashi[4], Tianyi Chen[1], Guizhi Shi[5], John Paul Shen[6], Scott Kopetz[6], Purushothama Rao Tata[4], Victor Moreno[7,8], Charles Gersbach[1], Gregory Crawford[9], David Hsu[10], Emina Huang[11], Pengcheng Bu[2,3,12] and Xiling Shen[1*]*

Chromatin Remodeling of Colorectal Cancer Liver Metastasis is Mediated by an HGF-PU.1-DPP4 Axis

Lihua Wang[1,#], Ergang Wang[1,#], Jorge Prado Balcazar[1], Zhenzhen Wu[2,3], Kun Xiang[1], Yi Wang[1], Qiang Huang[1], Marcos Negrete[1], Kai-Yuan Chen[1], Wei Li[1], Yujie Fu[1], Anders Dohlman[1], Robert Mines[1], Liwen Zhang[2,3], Yoshihiko Kobayashi[4], Tianyi Chen[1], Guizhi Shi[5], John Paul Shen[6], Scott Kopetz[6], Purushothama Rao Tata[4], Victor Moreno[7,8], Charles Gersbach[1], Gregory Crawford[9], David Hsu[10], Emina Huang[11], Pengcheng Bu[2,3,12*] and Xiling Shen[1*]

1. Department of Biomedical Engineering, Duke University, United States
2. Key Laboratory of RNA Biology, Key Laboratory of Protein and Peptide Pharmaceutical, Institute of Biophysics, Chinese Academy of Sciences, Beijing 100101, China
3. University of Chinese Academy of Sciences, Beijing 100049, China
4. Department of Cell Biology, Regeneration Next, Duke University School of Medicine, United States
5. Laboratory Animal Research Center, Institute of Biophysics, Chinese Academy of Sciences, Beijing, 100101, China
6. Department of Gastrointestinal Medical Oncology, MD Anderson, United States
7. Department of Clinical Sciences, University of Barcelona, Barcelona 08193, Spain. Cancer
8. Prevention and Control Program, Catalan Institute of Oncology-IDIBELL, CIBERESP, Barcelona E08907, Spain
9. Department of Pediatrics, Duke University School of Medicine, United States
10. Department of Medicine, Duke University School of Medicine, United States
11. Department of Cancer Biology and Colorectal Surgery, Lerner Research Institute, Cleveland Clinic, Cleveland, OH, 44195, United States
12. Center for Excellence in Biomacromolecules, Chinese Academy of Sciences, Beijing, 100101, China

\# These authors contributed equally.

* Correspondence: P.B. (bupc@ibp.ac.cn) and X.S. (xiling.shen@duke.edu)

Movie S1-2. Neutrophil mediated CRC cell killing in DPP4 KO (S1) or WT (S2) CRC cells.

Figure S1

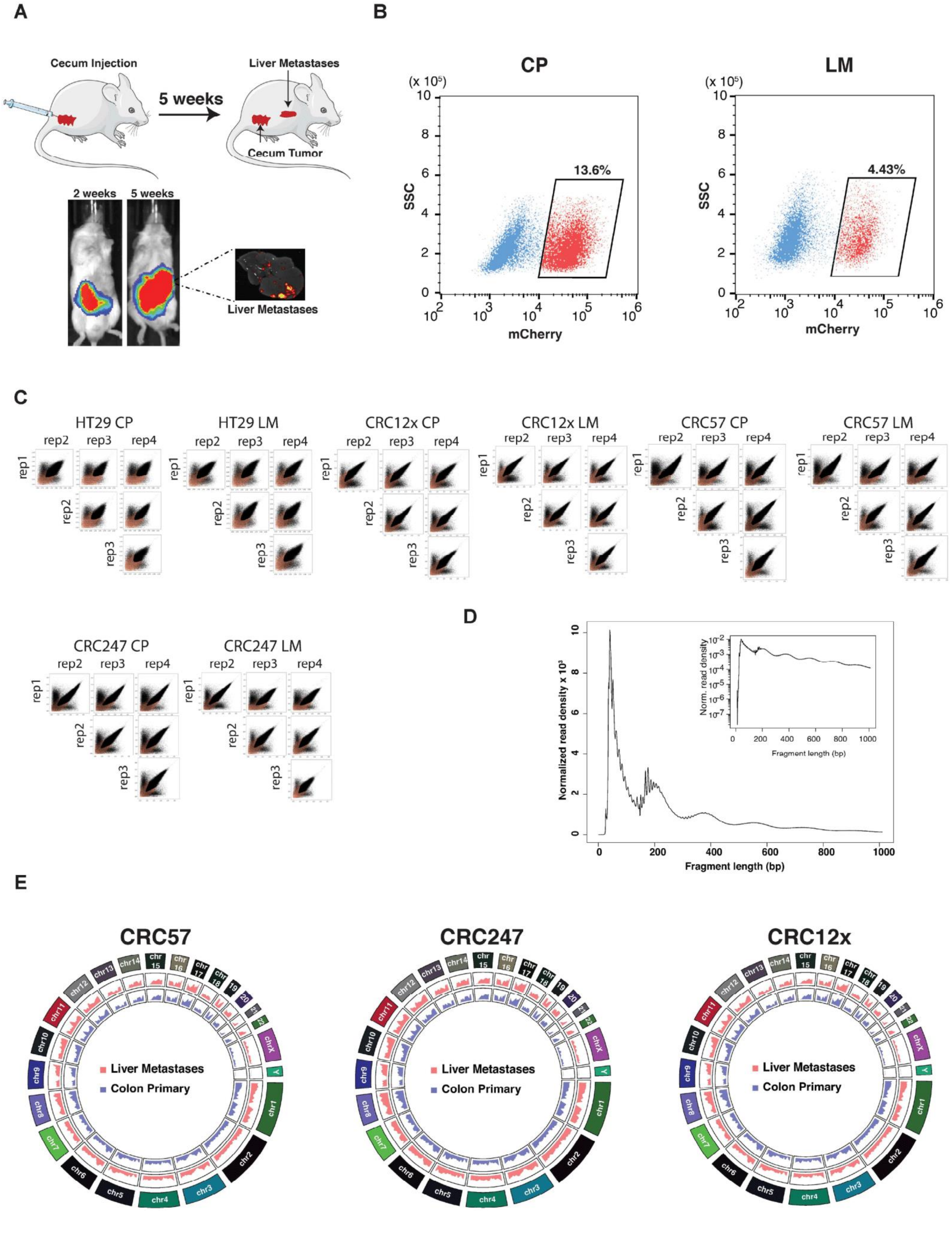
A
Cecum Injection
5 weeks
Liver Metastases
Cecum Tumor
2 weeks
5 weeks
Liver Metastases
B
CP
LM
(x 10^5)
SSC
mCherry
13.6%
4.43%
C
HT29 CP
HT29 LM
CRC12x CP
CRC12x LM
CRC57 CP
CRC57 LM
CRC247 CP
CRC247 LM
rep1
rep2
rep3
rep4
D
Normalized read density x 10^3
Fragment length (bp)
Norm. read density
E
CRC57
CRC247
CRC12x
Liver Metastases
Colon Primary

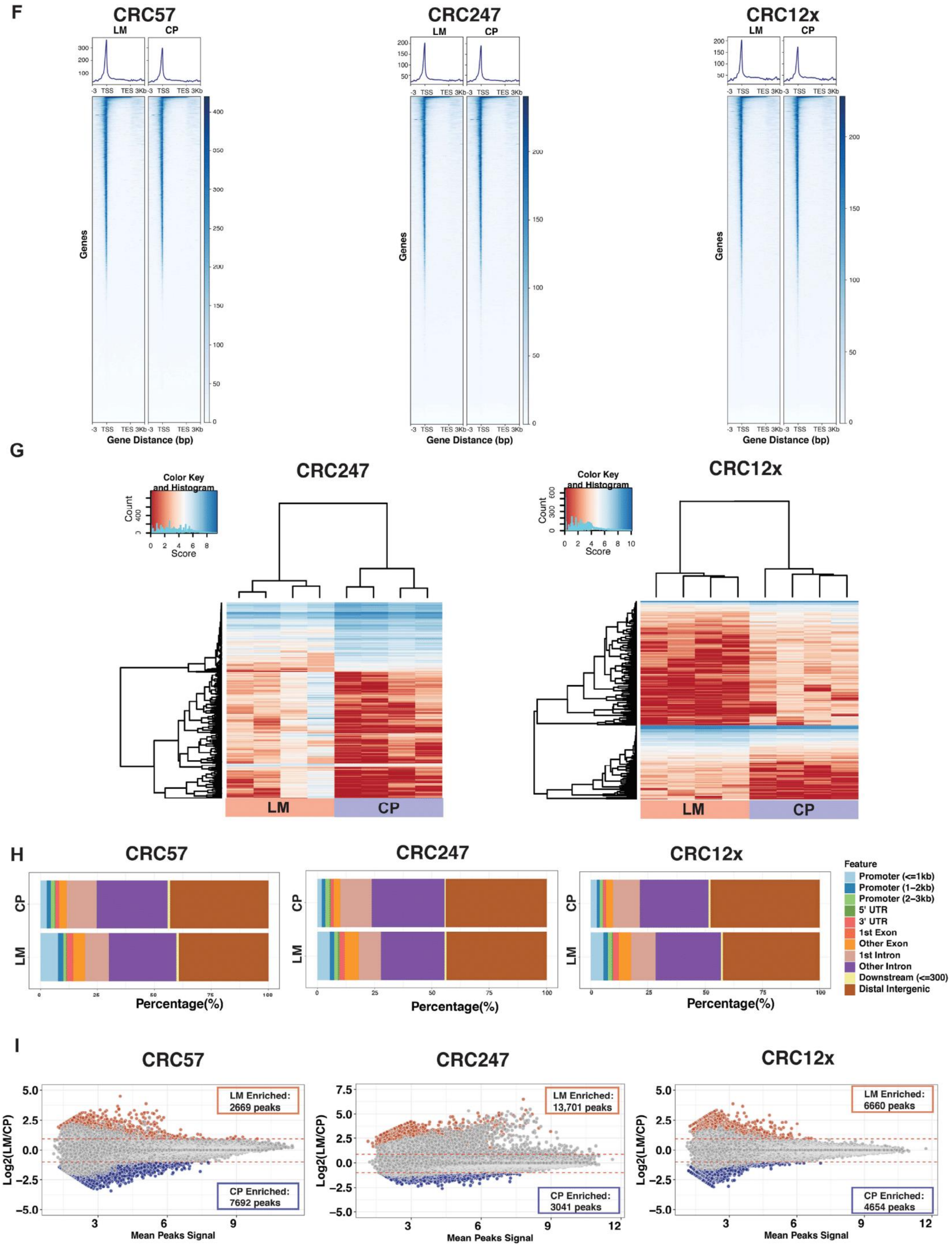
F
CRC57
CRC247
CRC12x
LM
CP
Genes
Gene Distance (bp)
G
Color Key and Histogram
Count
Score
LM
CP
H
Feature
Promoter (<=1kb)
Promoter (1–2kb)
Promoter (2–3kb)
5' UTR
3' UTR
1st Exon
Other Exon
1st Intron
Other Intron
Downstream (<=300)
Distal Intergenic
Percentage(%)
I
Log2(LM/CP)
Mean Peaks Signal
LM Enriched: 2669 peaks
CP Enriched: 7692 peaks
LM Enriched: 13,701 peaks
CP Enriched: 3041 peaks
LM Enriched: 6660 peaks
CP Enriched: 4654 peaks

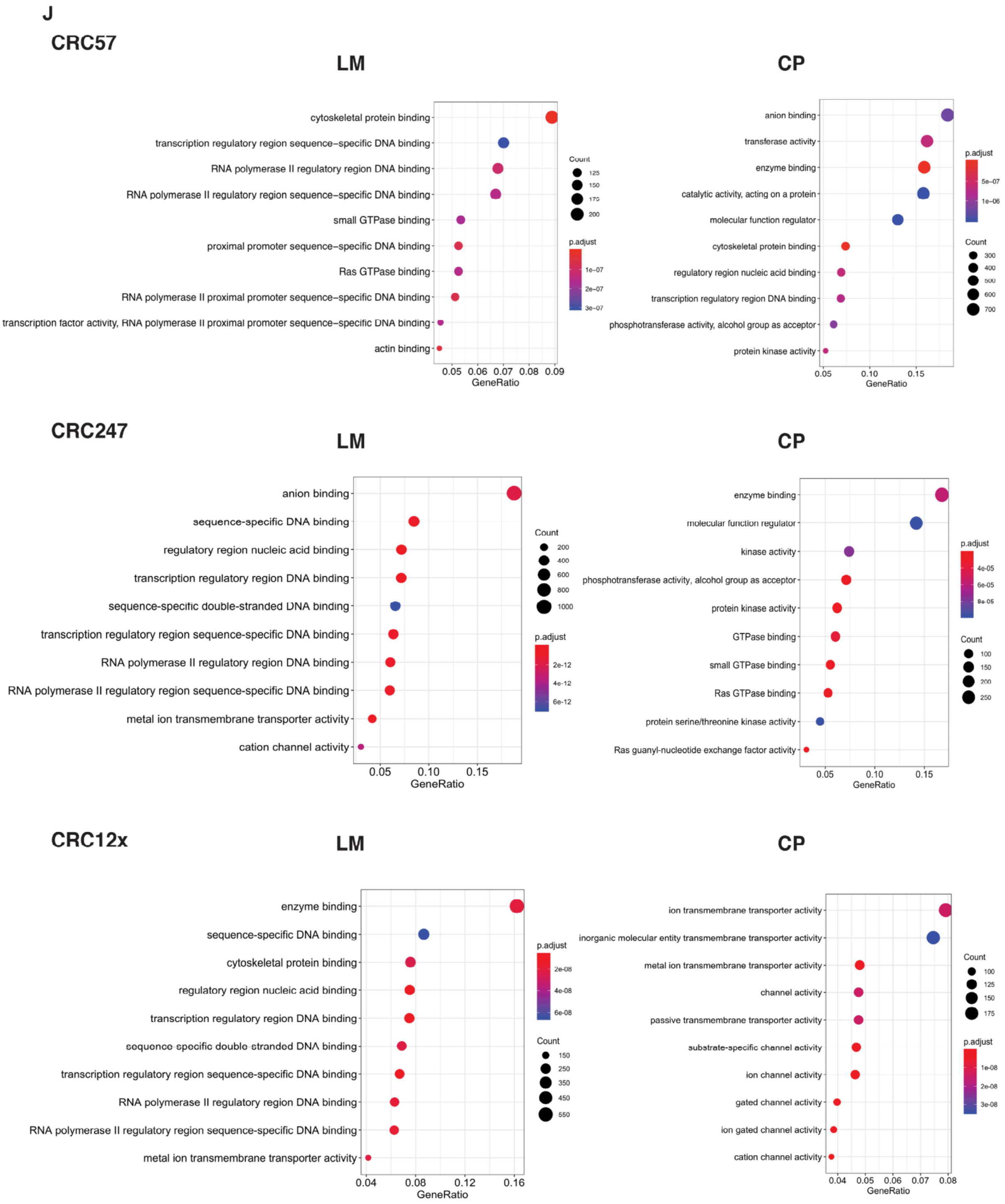

Figure S1. Epigenetic profiling on CRC liver metastases and CRC primary tumor

(**A**) Schematic and representative IVIS luciferase *in vivo* images of the orthotopic-metastatic cecum injection mouse model. (**B**) Representative FACS gating strategy for isolating mCherry-labeled CRC cells. (**C**) Irreproducible discovery rate (IDR) plots showing the correlation between four biological replicates for primary CRC and liver metastases in the ATAC-seq profiling of HT29, CRC12x, CRC57, and CRC247. (**D**) Representative fragment length/insert size distribution plot of an ATAC-seq library. (**E**) Circos plots revealing global chromatin accessibility changes across different chromosomes for primary CRC and liver

metastases in CRC57, CRC247, and CRC12x. (**F**) Heatmap showing chromatin accessibility across gene body from TSS to TES with 3 kb flanking regions for primary CRC and liver metastases in CRC57, CRC247, and CRC12x. (**G**) Hierarchical heatmap showing 1000 chromatin accessible regions with the highest variance between primary CRC and liver metastases in CRC247 and CRC12x across four biological replicates. (**H**) Distribution of enriched chromatin accessible regions across the genome for primary CRC and liver metastases in CRC57, CRC247, and CRC12x. (**I**) MA-plot showing the differentially enriched accessible chromatin regions for primary CRC vs. liver metastases in CRC57, CRC247, and CRC12x. (**J**) Gene Ontology (GO) analysis on molecular functions for differentially enriched accessible chromatin regions in CRC57, CRC247, and CRC12x primary CRC vs. liver metastases. LM, liver metastases. CP, CRC primary tumor.

Figure S2

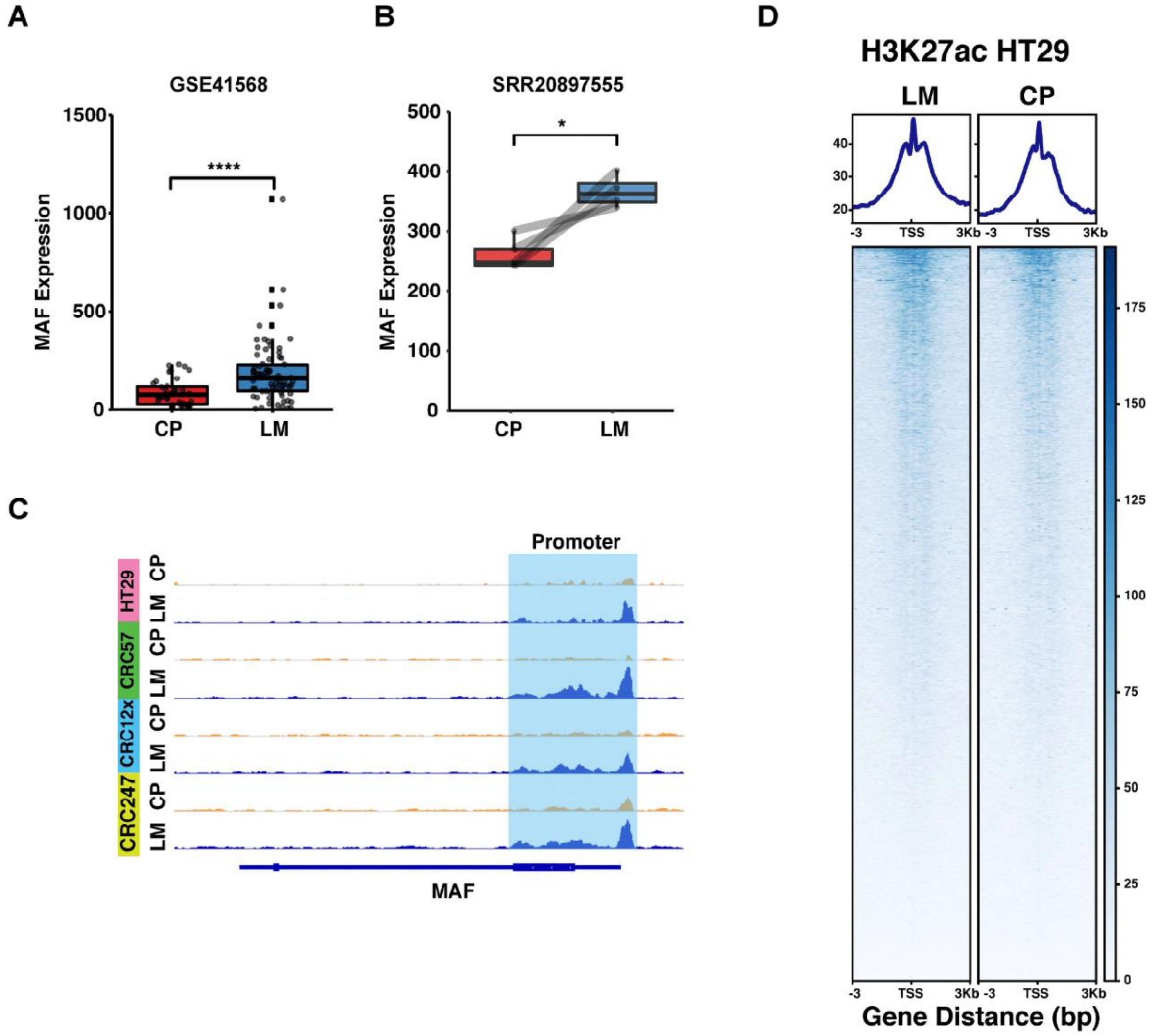

Figure S2. Analysis of MAF expression in primary CRC and liver metastases

(**A**) Analysis of differential MAF expression in GEO dataset (GSE41568) between primary CRC and liver metastases. (**B**) Analysis of differential MAF expression in the clinical RNA-seq dataset SRR2089755 from five matched patient liver metastases vs. primary CRC tumors. (**C**) ATAC-seq signal track showing MAF-associated open chromatin in liver metastases vs. primary CRC tumors. (**D**) Heatmap of H3K27 Mint-ChIP in HT29 liver metastases and primary CRC tumors showing the H3K27ac enrichment across gene body from TSS to TES with 3 kb flanking regions. LM, liver metastases. CP, CRC primary tumor. Data represent the mean ± s.d. in (A and B). p-values were calculated based on Student's t-test. *, $p<0.05$; **, $p<0.01$; ***, $p<0.001$; ****, $p<0.0001$.

Figure S3

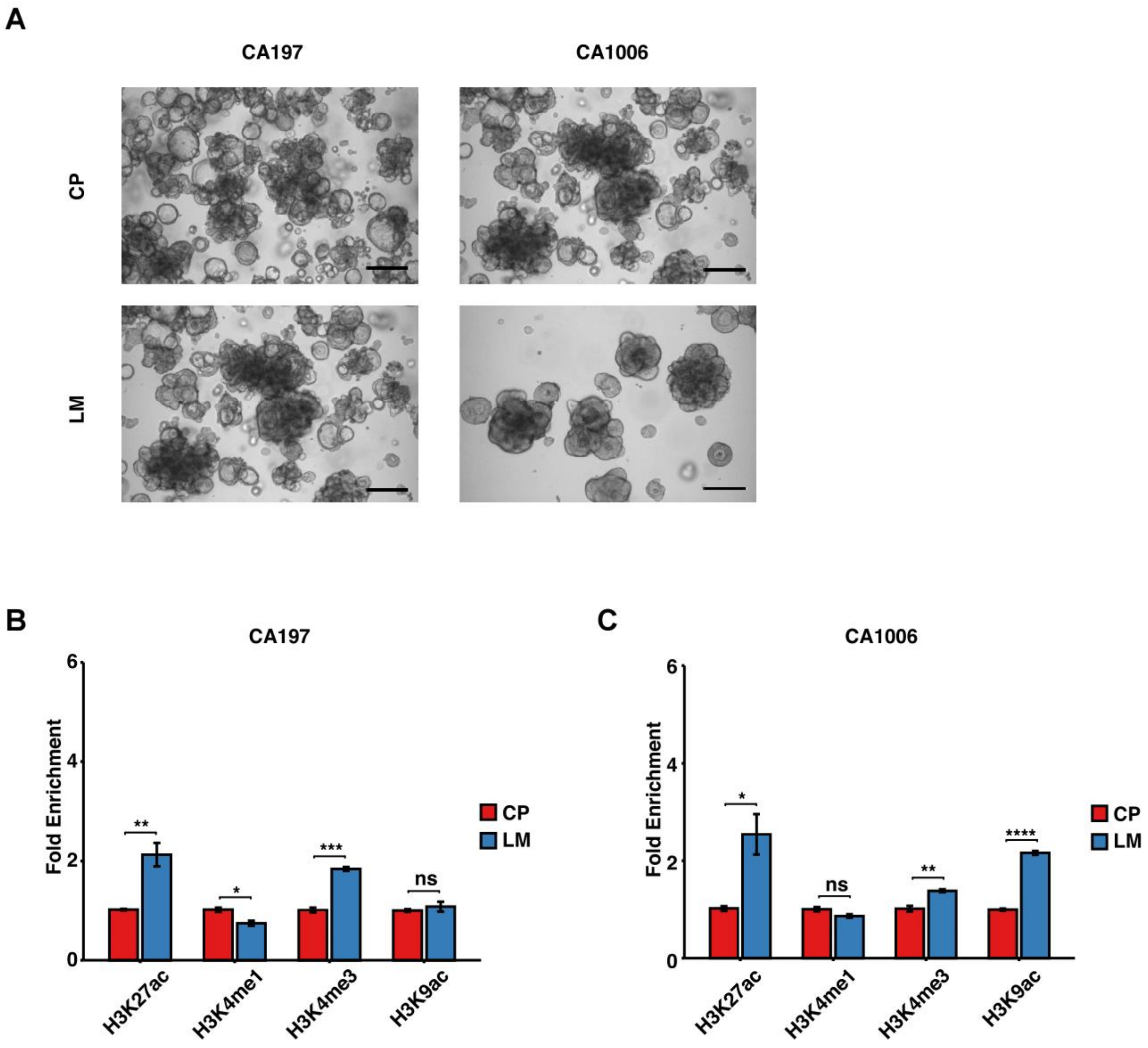

Figure S3. Epigenetic modification of DPP4 promoter in CRC liver metastasis

(**A**) Representative figures for two paired patient-derived organoids, CA197 and CA1006. (scale bar, 500 um) **(B and C)** ChIP-qPCR showing relative H3K27ac, H3K4me1, H3K4me3, and H3K9ac enrichments in primary CRC vs. liver metastases from CA197 or CA1006 patient-derived organoid. LM, liver metastases. CP, CRC primary tumor. Data represent the mean ± s.d. in (B and C). p-values were calculated based on Student's t-test. *, $p<0.05$; **, $p<0.01$; ***, $p<0.001$; ****, $p<0.0001$.

Figure S4

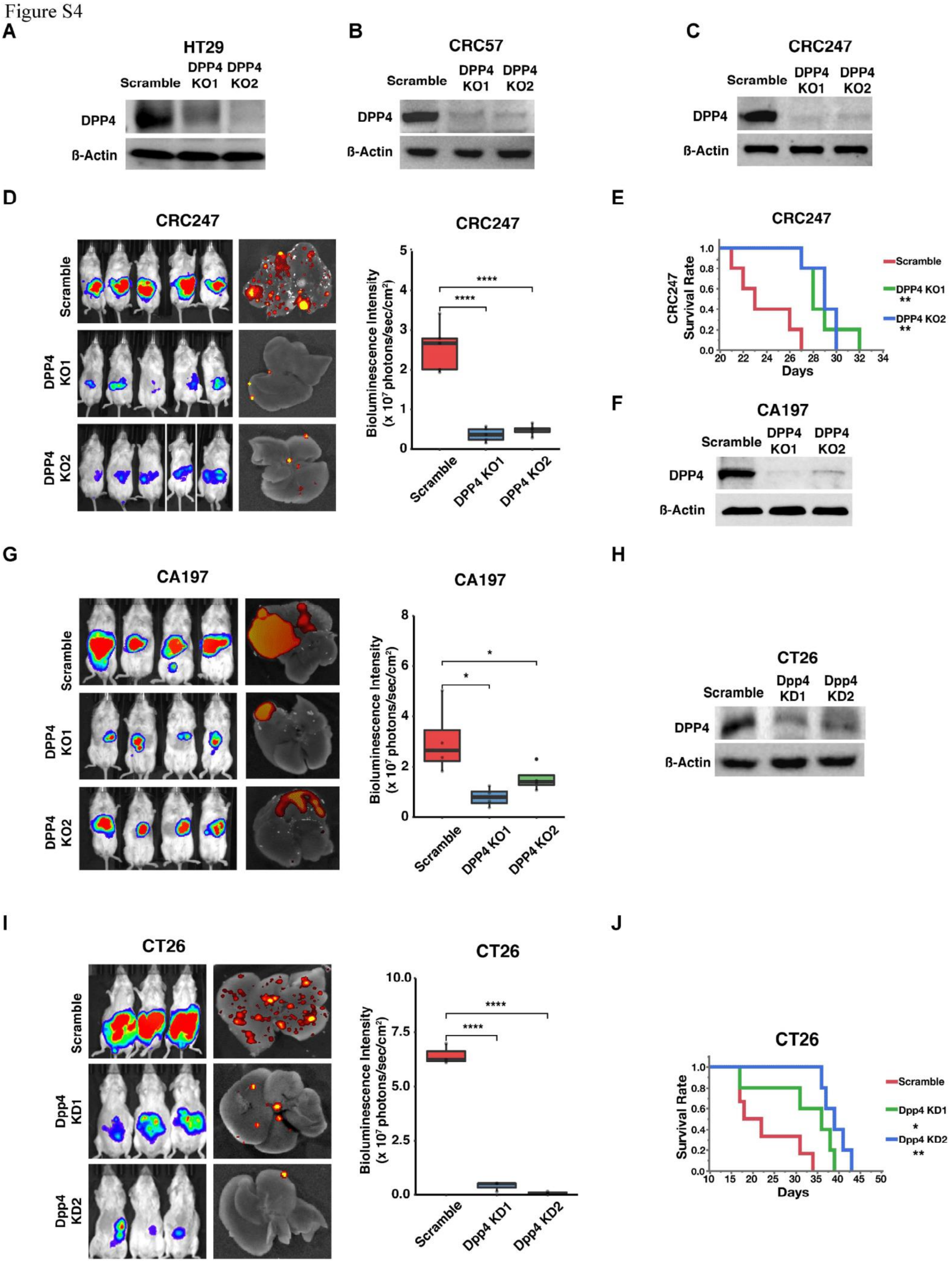

Figure S4. Downregulation of DPP4 suppresses CRC liver metastasis

(**A-C**) Western blots showing DPP4 expression levels in HT29 (A), CRC57 (B), and CRC247 (C) cells carrying scrambled (control) or DPP4 knockout (DPP4 KO1 and KO2) gRNA constructs. (**D** and **E)** Images and quantifications of bioluminescences (D) and survival analysis

(E) of NSG mice injected with luciferase-labeled CRC247 cells carrying scrambled (control) or DPP4 knockout (DPP4 KO1 or KO2) gRNA constructs. (**F**) Western blots showing DPP4 expression levels in CRC organoid CA197 cells carrying scrambled (control) or DPP4 knockout (DPP4 KO1 and KO2) gRNA constructs. (**G)** Images and quantifications of bioluminescences of NSG mice injected with luciferase-labeled CRC organoid CA197 cells carrying scrambled (control) or DPP4 knockout (DPP4 KO1 or KO2) gRNA constructs. (**H**) Western blots showing mouse *Dpp4* expression levels in CT26 carrying scrambled (control) or *Dpp4* knockdown (*Dpp4* KD1 and KD2) shRNA constructs. (**I** and **J)** Images and quantifications of bioluminescences (I) and survival analysis (J) of BALB/c immune competent mice injected with luciferase-labeled CT26 cells carrying scrambled (control) or *Dpp4* knockdown (*Dpp4* KD1 or KD2) shRNA constructs. Data represent the mean ± s.d. p-value was calculated based on ANOVA and Tukey's HSD post hoc test in (D), (G), and (I), and log-rank test in (E) and (J). *, p<0.05; **, p<0.01; ***, p<0.001; ****, p<0.0001.

Figure S5

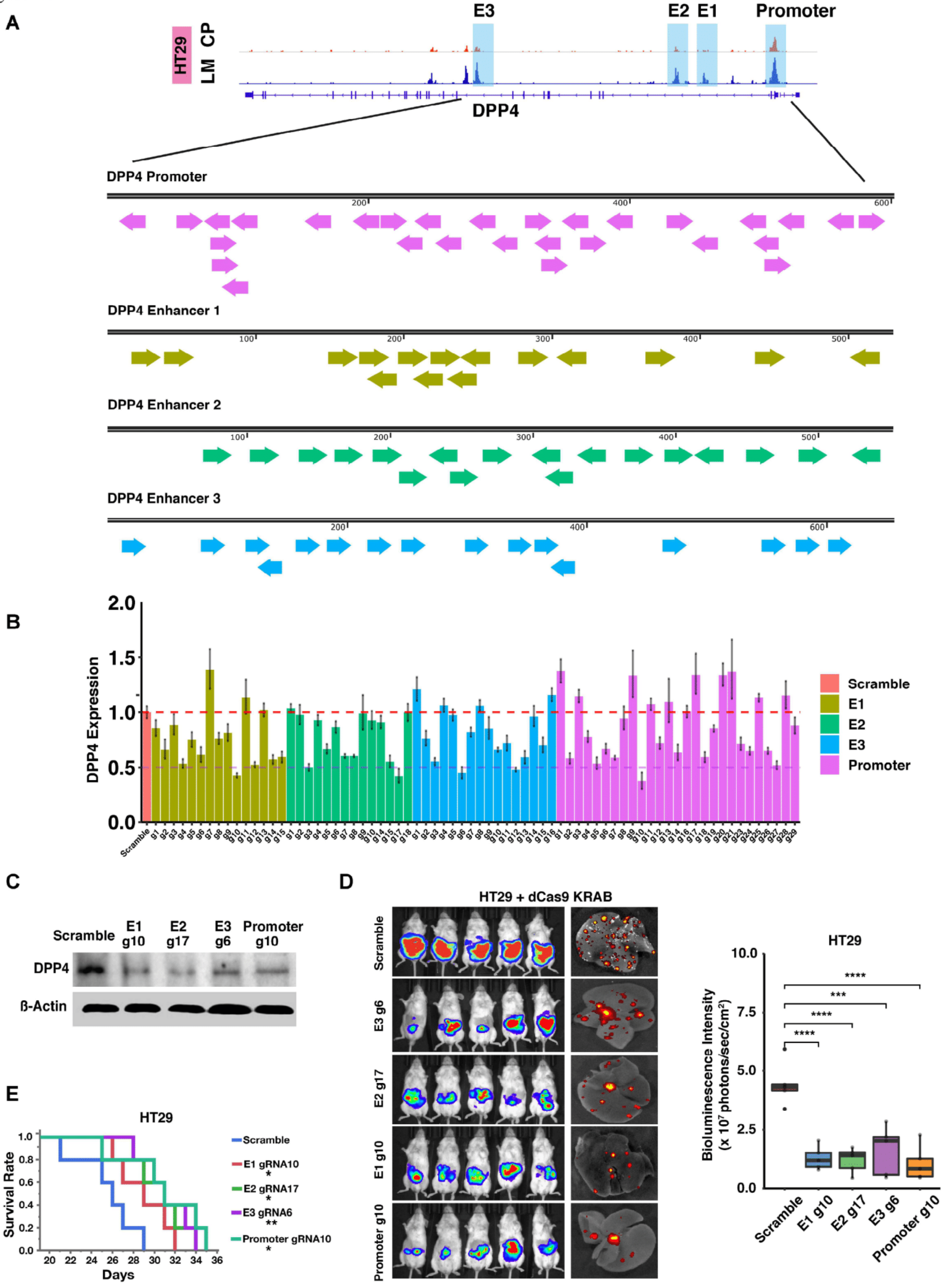
A
E3
E2 E1
Promoter
HT29
LM CP
DPP4
DPP4 Promoter
DPP4 Enhancer 1
DPP4 Enhancer 2
DPP4 Enhancer 3
B
DPP4 Expression
Scramble
E1
E2
E3
Promoter
C
Scramble
E1 g10
E2 g17
E3 g6
Promoter g10
DPP4
ß-Actin
D
HT29 + dCas9 KRAB
Scramble
E3 g6
E2 g17
E1 g10
Promoter g10
HT29
Bioluminescence Intensity (x 10^7 photons/sec/cm^2)
E
HT29
Survival Rate
Days
Scramble
E1 gRNA10
E2 gRNA17
E3 gRNA6
Promoter gRNA10

F

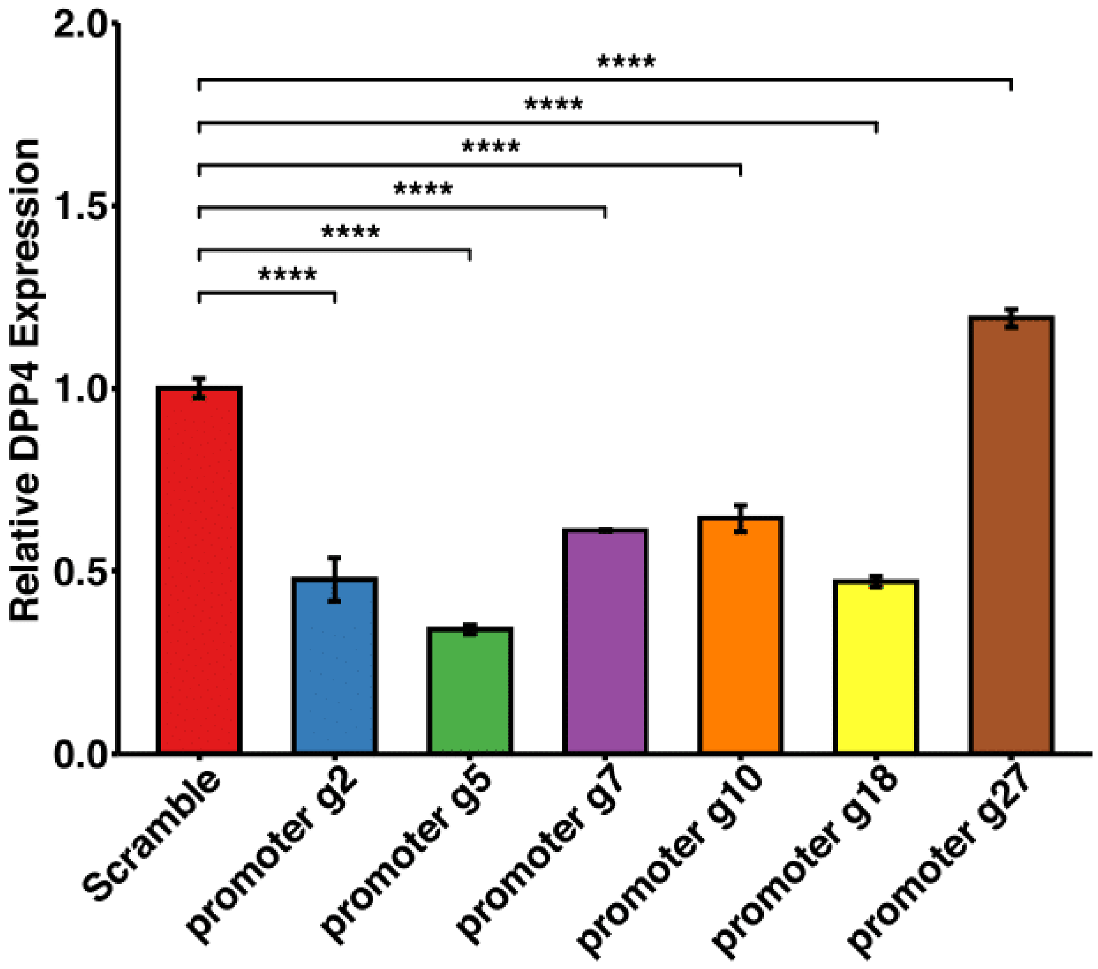

Figure S5. Epigenetic silencing of DPP4 via CRISPR/dCas9^{KRAB} suppresses CRC liver metastasis.

(**A**) Schematic design of gRNAs in the CRISPR/dCas9^{KRAB} screening on DPP4 promoter and three enhancers. (**B**) RT-qPCR screening of DPP4 expression in HT29 carrying the scrambled (control) or the designed gRNA constructs for CRISPR/dCas9^{KRAB} system. (**C**) Western blots showing downregulation of DPP4 by selected and scrambled gRNAs tested in the CRISPR/dCas9^{KRAB} system. (**D** and **E**) Images and quantification of bioluminescence (D) and survival analysis (E) of NSG mice carrying scrambled and selected gRNAs in the CRISPR/dCas9^{KRAB} system. (**F**) RT-qPCR screening of DPP4 expression in HT29 carrying the scrambled (control) or the designed gRNA constructs for CRISPR/dCas9^{HDAC} system. E1, E2, and E3, enhancer 1, enhancer 2, and enhancer 3. Data represent the mean ± s.d. p-values were calculated based on ANOVA and Tukey's HSD post hoc test in (D) and (F), and log-rank test in (E). *, $p<0.05$; **, $p<0.01$; ***, $p<0.001$; ****, $p<0.0001$.

Figure S6

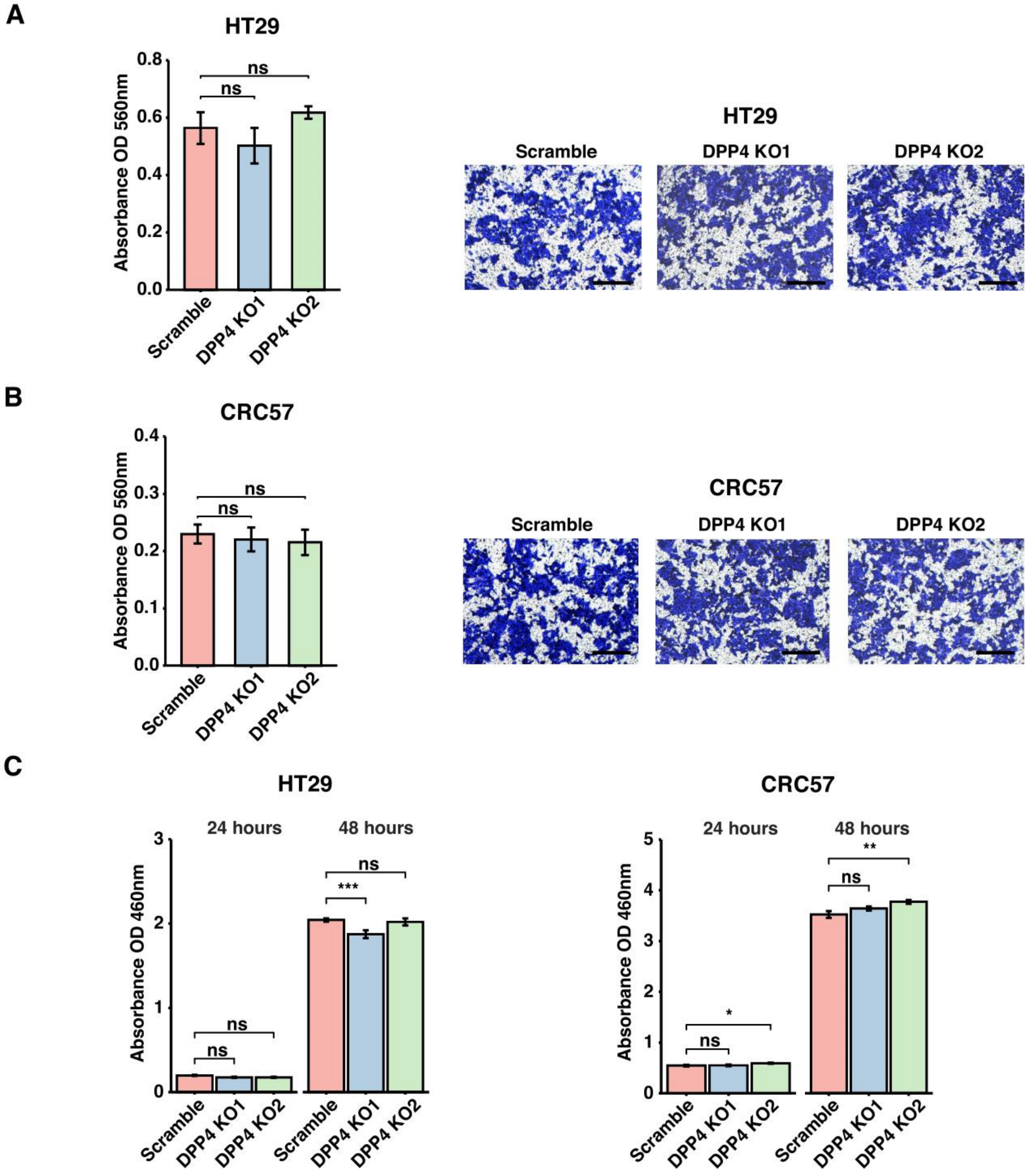

Figure S6. Migration and proliferation assay on DPP4 Knockout cells.
(A and B) Cell migration assay in HT29 or CRC57 cells carrying scrambled (control) or DPP4 knockout (DPP4 KO1 and KO2) gRNA constructs. **(C)** Cell proliferation assay in HT29 or CRC57 cells carrying scrambled (control) or DPP4 knockout (DPP4 KO1 and KO2) gRNA constructs at 24 hours and 48 hours. Data represent the mean ± s.d. p-values were calculated based on ANOVA and Tukey's HSD post hoc test. *, $p<0.05$; **, $p<0.01$; ***, $p<0.001$; ****, $p<0.0001$.

Figure S7

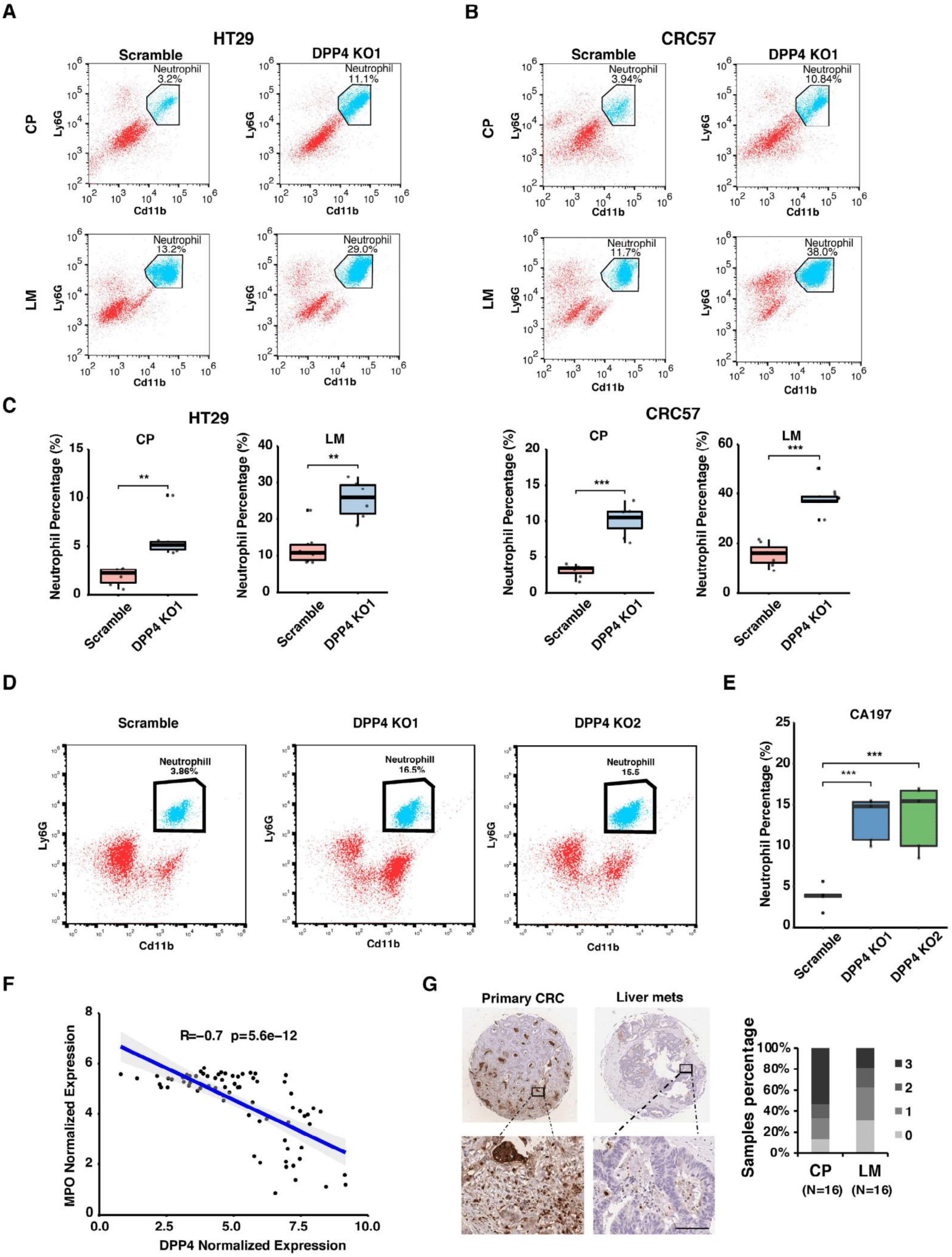
A
HT29
Scramble
DPP4 KO1
CP
LM
Ly6G
Cd11b
Neutrophil 3.2%
Neutrophil 11.1%
Neutrophil 13.2%
Neutrophil 29.0%
B
CRC57
Scramble
DPP4 KO1
Neutrophil 3.94%
Neutrophil 10.84%
Neutrophil 11.7%
Neutrophil 38.0%
C
HT29
CRC57
CP
LM
Neutrophil Percentage (%)
Scramble
DPP4 KO1
D
Scramble
DPP4 KO1
DPP4 KO2
Neutrophill 3.86%
Neutrophill 16.5%
Neutrophill 15.5
E
CA197
Neutrophil Percentage (%)
Scramble
DPP4 KO1
DPP4 KO2
F
R=−0.7 p=5.6e−12
MPO Normalized Expression
DPP4 Normalized Expression
G
Primary CRC
Liver mets
Samples percentage
CP (N=16)
LM (N=16)

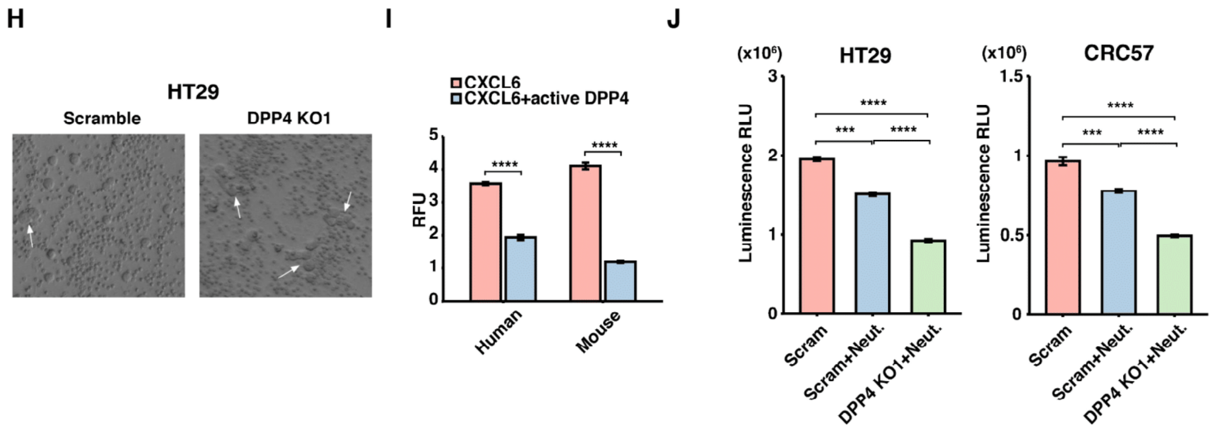

Figure S7. Ablation of DPP4 facilitates recruitment of neutrophils

(**A-C**) Representative FACS plots (A and B) and quantification (C) of neutrophil levels from the liver metastases and primary CRC tumors of NSG mice injected with HT29 or CRC57 carrying scrambled (control) or DPP4 knockout (DPP4 KO1 or KO2) gRNA constructs. (**D** and **E**) Representative FACS plots (D) and quantification (E) of neutrophil levels from the liver metastases of NSG mice injected with CRC organoid cell CA197 carrying scrambled (control) or DPP4 knockout (DPP4 KO1 or KO2) gRNA constructs. (**F**) Integrated analysis of GEO datasets (GSE40367, GSE41258 and GSE49355) showing the reverse correlation of Myeloperoxidase (MPO, neutrophil marker gene) and DPP4 expression in CRC liver metastases. (**G**) Representative IHC staining and evaluation of PU.1 expression measured on a tissue microarray that contains 16 paired primary CRC and liver metastases. (Scale bar, 50 um). (**H**) Images from time-lapse microscopy showing the co-culture of HT29 cells carrying scrambled (control) or DPP4 KO1 gRNA construct and tumor-associated neutrophils. White-arrow, neutrophil mediated tumor cell killing. (**I**) Trans-well assay showing neutrophil trafficking with innate or DPP4-truncated human or mouse CXCL6. (**J**) Luminescence signal showing viability of HT29 or CRC57 cells carrying scrambled (control) or DPP4 KO1 gRNA constructs with or without tumor-associated neutrophils Scram, scrambled gRNA. Neut., neutrophils. LM, liver metastases. CP, CRC primary tumor. Data represent the mean ± s.d. p-values were calculated based on Student's t-test in (C) and (I), and ANOVA and Tukey's HSD post hoc test in (E) and (J). *, $p<0.05$; **, $p<0.01$; ***, $p<0.001$; ****, $p<0.0001$.

Figure S8

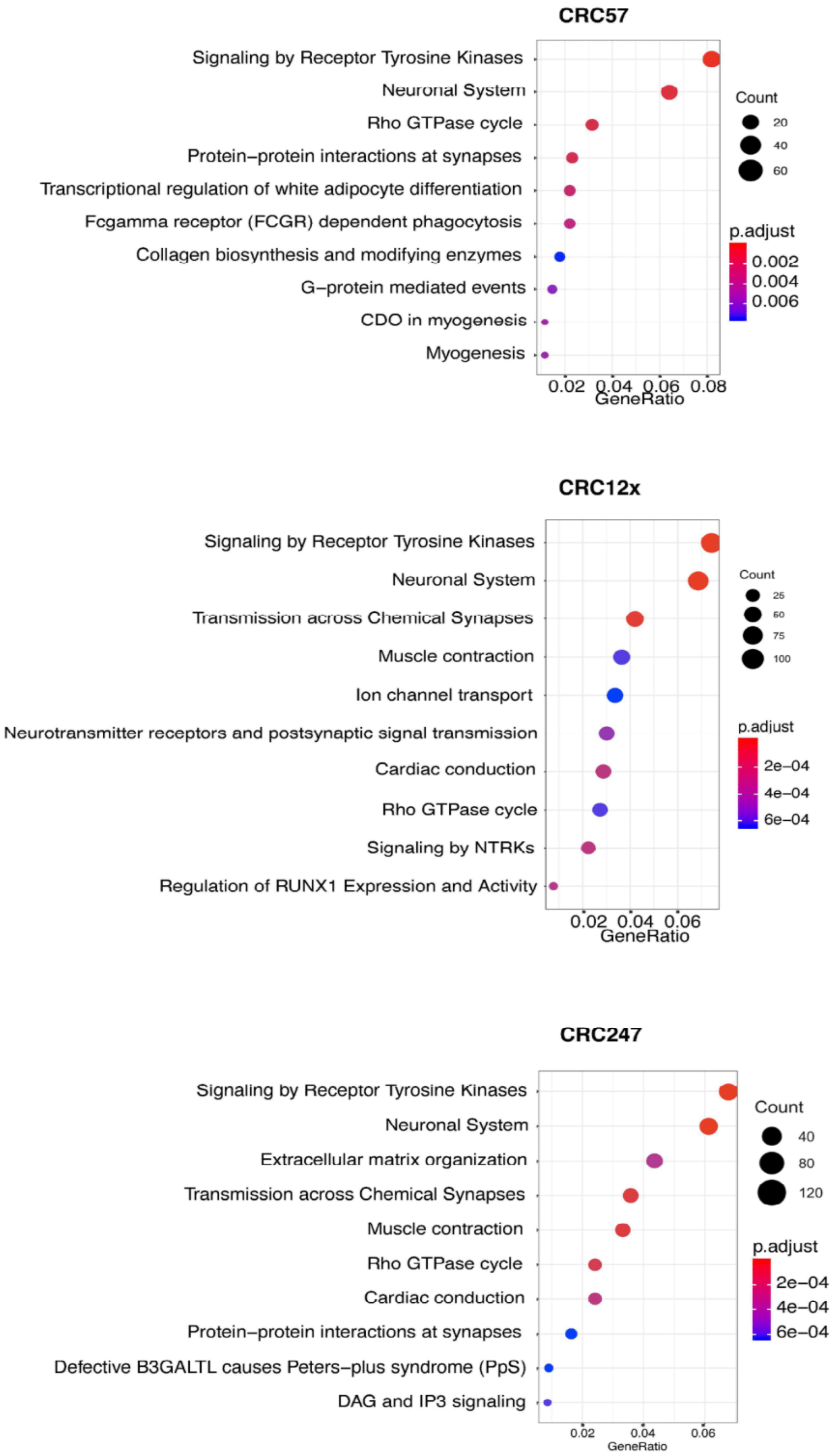

Figure S8. Dotplot of pathway analysis on enriched peaks in CRC liver metastases.
(A-C) Dotplot of GO Reactome pathway analysis on enriched peaks in CRC liver metastases from the ATAC-seq results of CRC57, CRC12x, and CRC247 cells.

Figure S9

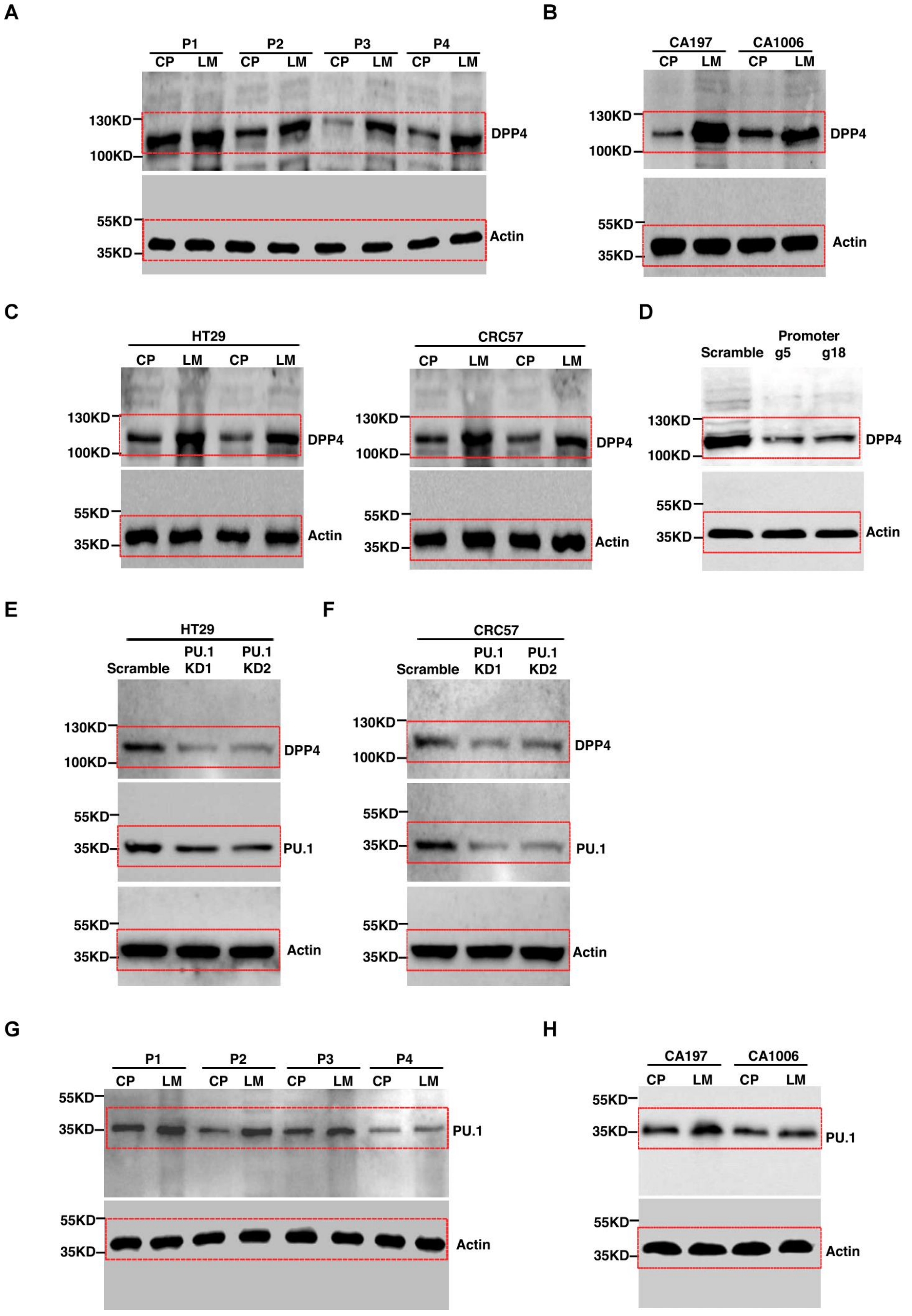
A
P1 P2 P3 P4
CP LM CP LM CP LM CP LM
130KD
100KD
DPP4
55KD
35KD
Actin
B
CA197 CA1006
CP LM CP LM
130KD
100KD
DPP4
55KD
35KD
Actin
C
HT29
CP LM CP LM
130KD
100KD
DPP4
55KD
35KD
Actin
CRC57
CP LM CP LM
130KD
100KD
DPP4
55KD
35KD
Actin
D
Promoter
Scramble g5 g18
130KD
100KD
DPP4
55KD
35KD
Actin
E
HT29
Scramble PU.1 KD1 PU.1 KD2
130KD
100KD
DPP4
55KD
35KD
PU.1
55KD
35KD
Actin
F
CRC57
Scramble PU.1 KD1 PU.1 KD2
130KD
100KD
DPP4
55KD
35KD
PU.1
55KD
35KD
Actin
G
P1 P2 P3 P4
CP LM CP LM CP LM CP LM
55KD
35KD
PU.1
55KD
35KD
Actin
H
CA197 CA1006
CP LM CP LM
55KD
35KD
PU.1
55KD
35KD
Actin

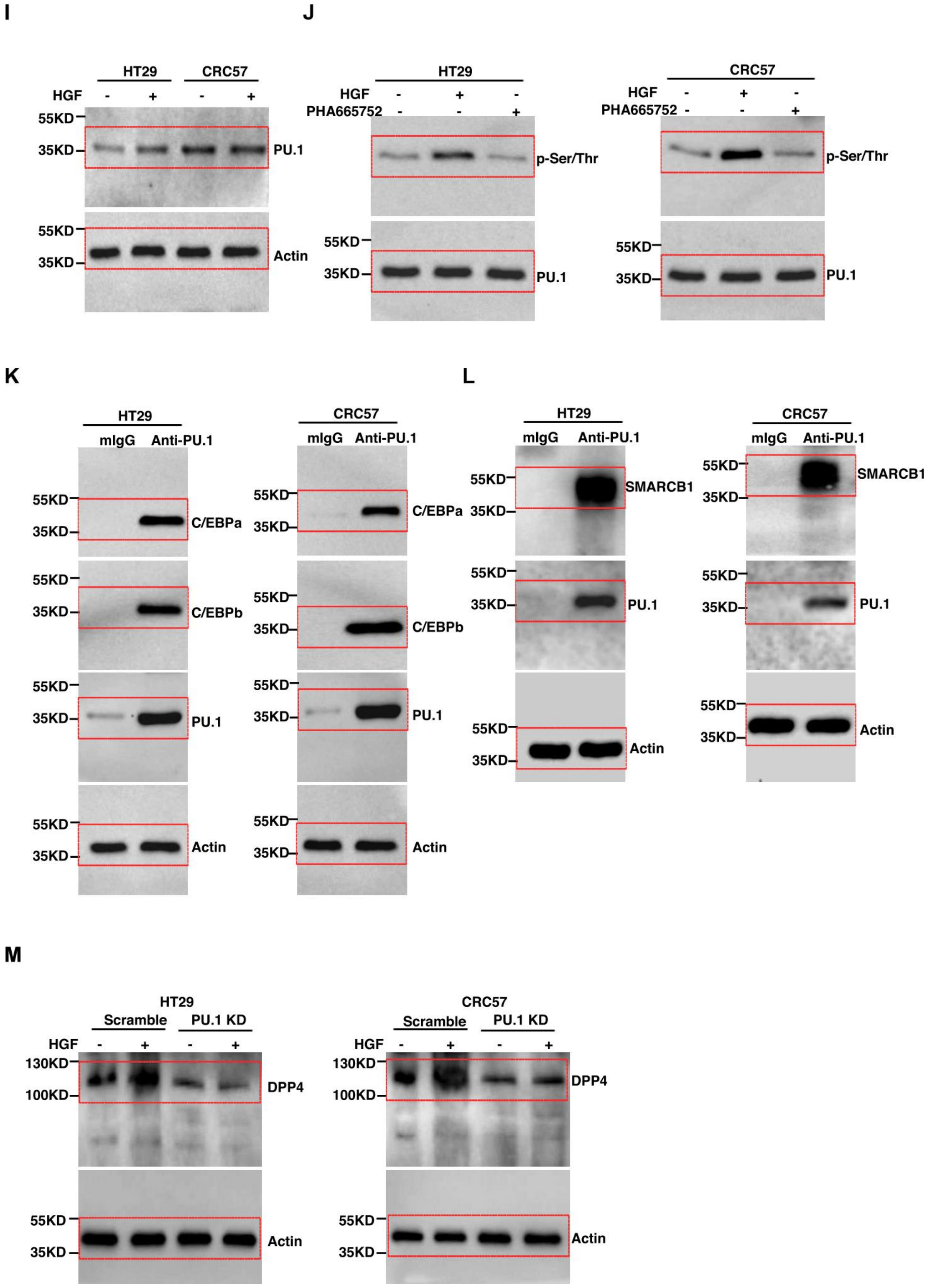

Figure S9. Full scans of Western blots in main figures.

(**A**-**C**) Full scans of Western blots for Figure 2H (A), 2J (B) and 2K (C). (**D**) Full scan of Western blot for Figure 3D. **(E** and **F)** Full scans of Western blots for Figure 4G. **(G** and **H)** Full scans of Western blots for Figure 5D (G) and 5E (H). **(I-M)** Full scans of Western blots for Figure 6B (I), 6C (J), 6D (K), 6E (L), 6G (M).

Figure S10

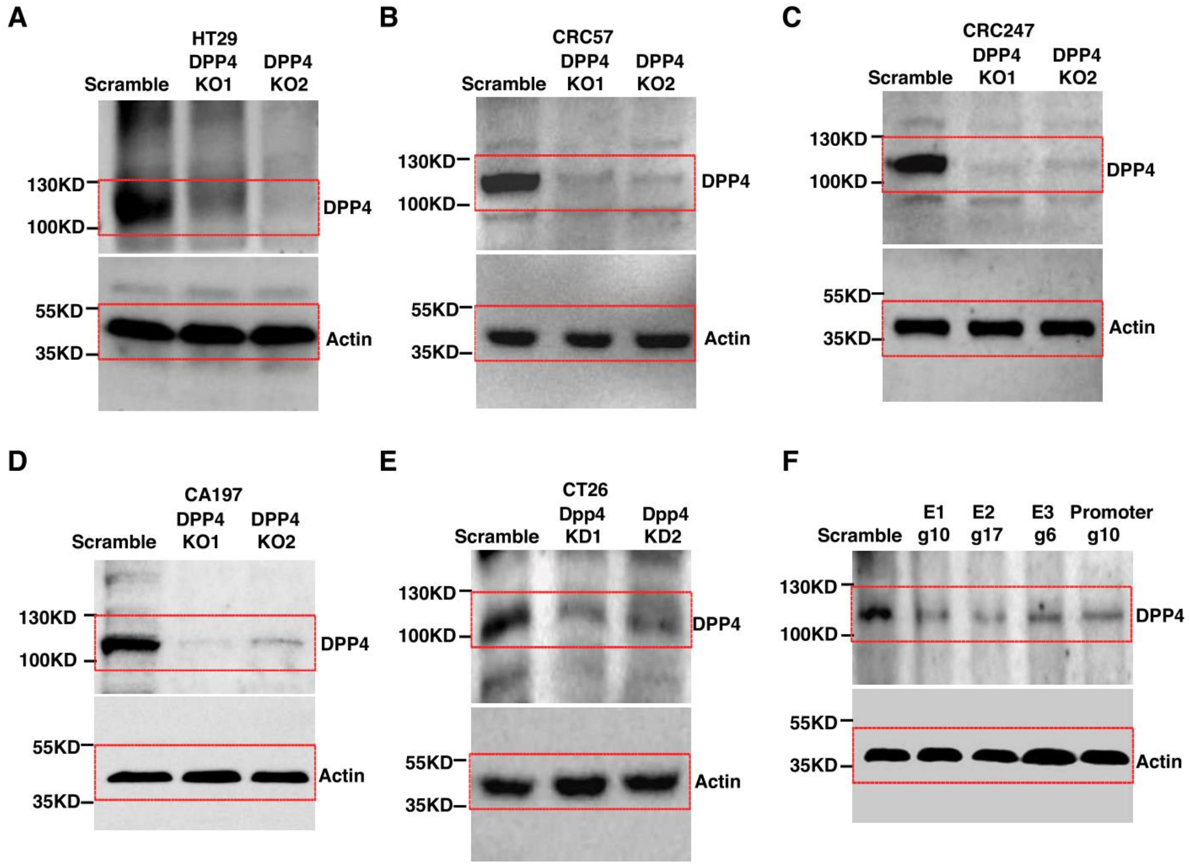

Figure S10. Full scans of Western blots in supplementary figures.
(A-E) Full scans of Western blots for Figure S4A (A), S4B (B), S4C (C), S4F (D) and S4H (E).
(F) Full scan of Western blot for Figure S5C.
