# Supplementary material for: Chromatin Remodeling of Colorectal Cancer Liver Metastasis is Mediated by an HGF‐PU.1‐DPP4 Axis
Source: Adv Sci (Weinh). 2021 Aug 10;8(19):2004673. doi: 10.1002/advs.202004673 (PMC8498885; doi:10.1002/advs.202004673)
Supplement: Supplementary file 2 — Supplemental Table 1‐4 [file ADVS-8-2004673-s005.pdf]

Table S1. Information for patient-derived xenograft cell lines.

Patients	Gender	Primary site	Metastatic site	Differentiation	Stage
CRC57	Male	Colon	Liver	Moderate	IV
CRC247	Male	Colon	Liver	Moderate	IV
CRC12x	Female	Colon	Liver	Moderate	IV

Table S2. Information of patients who provided tissues for RT-qPCR.

Patients	Age	Gender	Primary site	Differentiation	Stage	Metastatic site
P1	64	Male	Ileocecum	Moderate	IV	Liver
P2	63	Male	Colon	Poor	IV	Liver
P3	73	Male	Colon	Moderate	IV	Liver
P4	69	Male	Colon	Poor	IV	Liver
P5	68	Male	Rectum	Poor	IV	Liver
P6	34	Male	Colon	Moderate	IV	Liver
P7	61	Female	Colon	Moderate	IV	Liver
P8	48	Female	Colon	Poor	IV	Liver
P9	59	Male	Colon	Poor	IV	Liver
P10	62	Female	Colon	Moderate	IV	Liver

Table S3. Information of patients who provided tissues for tumor array.

Patients	Age	Gender	Primary site	Differentiation	Stage
D15A0311	53	Female	Colon	Moderate	IV
D15A0447	-	Female	Colon	Moderate	IV
D15A0643	61	Male	Colon	Poorly	IV
D15A1724	60	Male	Colon	Moderate	IV
D15A3732	73	Male	Colon	Moderate	IV
D15A0277	61	Male	Colon	Moderate	IV
D15A3468	73	Male	Colon	Well	IV
D16A0417	46	Male	rectum	Moderate	IV
D16A0492	67	Male	rectum	Moderate	IV
D16A1332	-	Male	rectum	Moderate	IV
D16A4335	59	Male	rectum	Poor	IV
D16A4836	59	Male	rectum	Poor	IV
D16A0763	53	Female	rectum	Moderate	IV
D16A0876	62	Male	rectum	Moderate	IV
D16A3519	66	Female	rectum	Moderate	IV
D16A3523	60	Male	rectum	Moderate	IV

Table S4. Information for patient-derived organoid.

Patients	Age	Gender	Primary site	Stage
CA197	46	Female	Colon	IV
CA1006	56	Male	Colon	IV
