# Supplementary material for: Chromatin Remodeling of Colorectal Cancer Liver Metastasis is Mediated by an HGF‐PU.1‐DPP4 Axis
Source: Adv Sci (Weinh). 2021 Aug 10;8(19):2004673. doi: 10.1002/advs.202004673 (PMC8498885; doi:10.1002/advs.202004673)
Supplement: Supplementary file 5 — Supplemental Table 7 [file ADVS-8-2004673-s001.docx]

**Table S7.1. Guide RNA sequences.**

| **Name** | **Sequences** |
| --- | --- |

| Enhancer1-sgRNA1-hU6-S | CACCGGCATTAGGCCGTGAAATCCG |
| --- | --- |
| Enhancer1-sgRNA1-hU6-AS | AAACCGGATTTCACGGCCTAATGCC |
| Enhancer1-sgRNA2-hU6-S | CACCGGGCCCAAACTAATACGTACA |
| Enhancer1-sgRNA2-hU6-AS | AAACTGTACGTATTAGTTTGGGCCC |
| Enhancer1-sgRNA3-hU6-S | CACCGAGCCCTGTACGTATTAGTTT |
| Enhancer1-sgRNA3-hU6-AS | AAACAAACTAATACGTACAGGGCTC |
| Enhancer1-sgRNA4-hU6-S | CACCGTATCATCACCTCGGATTTCA |
| Enhancer1-sgRNA4-hU6-AS | AAACTGAAATCCGAGGTGATGATAC |
| Enhancer1-sgRNA5-hU6-S | CACCGCTATGCCCCATGAAATTGAC |
| Enhancer1-sgRNA5-hU6-AS | AAACGTCAATTTCATGGGGCATAGC |
| Enhancer1-sgRNA6-hU6-S | CACCGTCAAGTGTTTATCATCACCT |
| Enhancer1-sgRNA6-hU6-AS | AAACAGGTGATGATAAACACTTGAC |
| Enhancer1-sgRNA7-hU6-S | CACCGTCAGGAAGCTCTACACACAC |
| Enhancer1-sgRNA7-hU6-AS | AAACGTGTGTGTAGAGCTTCCTGAC |
| Enhancer1-sgRNA8-hU6-S | CACCGGGAGCCAGCACTAAGCAAGG |
| Enhancer1-sgRNA8-hU6-AS | AAACCCTTGCTTAGTGCTGGCTCCC |
| Enhancer1-sgRNA9-hU6-S | CACCGGATTACACTTATAAATCTTC |
| Enhancer1-sgRNA9-hU6-AS | AAACGAAGATTTATAAGTGTAATCC |
| Enhancer1-sgRNA10-hU6-S | CACCGTGTATGACTAAATGACTCAC |
| Enhancer1-sgRNA10-hU6-AS | AAACGTGAGTCATTTAGTCATACAC |
| Enhancer1-sgRNA11-hU6-S | CACCGTTGCAGTGAGACTTCAGTGG |
| Enhancer1-sgRNA11-hU6-AS | AAACCCACTGAAGTCTCACTGCAAC |
| Enhancer1-sgRNA12-hU6-S | CACCGAAACAAGACTGATACAATAA |
| Enhancer1-sgRNA12-hU6-AS | AAACTTATTGTATCAGTCTTGTTTC |
| Enhancer1-sgRNA13-hU6-S | CACCGGTAAAGGAAATCTGCATTTA |
| Enhancer1-sgRNA13-hU6-AS | AAACTAAATGCAGATTTCCTTTACC |
| Enhancer1-sgRNA14-hU6-S | CACCGCCTAATGCCAGTCAATTTCA |
| Enhancer1-sgRNA14-hU6-AS | AAACTGAAATTGACTGGCATTAGGC |
| Enhancer1-sgRNA15-hU6-S | CACCGAAAATAGTTGCATGTTTCAC |
| Enhancer1-sgRNA15-hU6-AS | AAACGTGAAACATGCAACTATTTTC |
| Enhancer2-sgRNA1-hU6-S | CACCGCTACGACTGTGCGGGTGTAC |
| Enhancer2-sgRNA1-hU6-AS | AAACGTACACCCGCACAGTCGTAGC |
| Enhancer2-sgRNA2-hU6-S | CACCGCTCTGAACTGTTACGACCCC |
| Enhancer2-sgRNA2-hU6-AS | AAACGGGGTCGTAACAGTTCAGAGC |
| Enhancer2-sgRNA3-hU6-S | CACCGGAGGGATTTGTCAGCGAACC |
| Enhancer2-sgRNA3-hU6-AS | AAACGGTTCGCTGACAAATCCCTCC |
| Enhancer2-sgRNA4-hU6-S | CACCGACAAAGAGCAACTGCAACCG |
| Enhancer2-sgRNA4-hU6-AS | AAACCGGTTGCAGTTGCTCTTTGTC |
| Enhancer2-sgRNA5-hU6-S | CACCGCAGTGAATCACCCGAGGAAG |
| Enhancer2-sgRNA5-hU6-AS | AAACCTTCCTCGGGTGATTCACTGC |
| Enhancer2-sgRNA6-hU6-S | CACCGGGATGGGGTATGTCATGAGC |
| Enhancer2-sgRNA6-hU6-AS | AAACGCTCATGACATACCCCATCCC |
| Enhancer2-sgRNA7-hU6-S | CACCGATGGGGATGCCAACCCTCCC |
| Enhancer2-sgRNA7-hU6-AS | AAACGGGAGGGTTGGCATCCCCATC |
| Enhancer2-sgRNA8-hU6-S | CACCGATGTTGCAGCTTTATAGGCC |
| Enhancer2-sgRNA8-hU6-AS | AAACGGCCTATAAAGCTGCAACATC |
| Enhancer2-sgRNA9-hU6-S | CACCGAACATCTGTTGAATGGCTCT |
| Enhancer2-sgRNA9-hU6-AS | AAACAGAGCCATTCAACAGATGTTC |
| Enhancer2-sgRNA10-hU6-S | CACCGGCTAGTGACATCTGCACATT |
| Enhancer2-sgRNA10-hU6-AS | AAACAATGTGCAGATGTCACTAGCC |
| Enhancer2-sgRNA11-hU6-S | CACCGATATCCTGTGTGACAATCTG |
| Enhancer2-sgRNA11-hU6-AS | AAACCAGATTGTCACACAGGATATC |
| Enhancer2-sgRNA12-hU6-S | CACCGTCAACTGGAAATCATATTGG |
| Enhancer2-sgRNA12-hU6-AS | AAACCCAATATGATTTCCAGTTGAC |
| Enhancer2-sgRNA13-hU6-S | CACCGTTATATCTATTGTGTCATCC |
| Enhancer2-sgRNA13-hU6-AS | AAACGGATGACACAATAGATATAAC |
| Enhancer2-sgRNA14-hU6-S | CACCGCCCTCAGGAGAGGAATGGGT |
| Enhancer2-sgRNA14-hU6-AS | AAACACCCATTCCTCTCCTGAGGGC |
| Enhancer2-sgRNA15-hU6-S | CACCGATGCCACCCTCTGCTCTGAT |
| Enhancer2-sgRNA15-hU6-AS | AAACATCAGAGCAGAGGGTGGCATC |
| Enhancer2-sgRNA16-hU6-S | CACCGATGAGATGAAGCAGATCTGA |
| Enhancer2-sgRNA16-hU6-AS | AAACTCAGATCTGCTTCATCTCATC |
| Enhancer2-sgRNA17-hU6-S | CACCGATACTGCCAGTGCTGGCCTC |
| Enhancer2-sgRNA17-hU6-AS | AAACGAGGCCAGCACTGGCAGTATC |
| Enhancer2-sgRNA18-hU6-S | CACCGACCTGCATGCTACGACTGTG |
| Enhancer2-sgRNA18-hU6-AS | AAACCACAGTCGTAGCATGCAGGTC |
| Enhancer3-sgRNA1-hU6-S | CACCGGTGGGGATAACCCTTATTCC |
| Enhancer3-sgRNA1-hU6-AS | AAACGGAATAAGGGTTATCCCCACC |
| Enhancer3-sgRNA2-hU6-S | CACCGCTTCTCACCTTGGACTACTC |
| Enhancer3-sgRNA2-hU6-AS | AAACGAGTAGTCCAAGGTGAGAAGC |
| Enhancer3-sgRNA3-hU6-S | CACCGAAGAAGGATGACTAATTGAC |
| Enhancer3-sgRNA3-hU6-AS | AAACGTCAATTAGTCATCCTTCTTC |
| Enhancer3-sgRNA4-hU6-S | CACCGTGGAGCCCCAGAGTAGTCCA |
| Enhancer3-sgRNA4-hU6-AS | AAACTGGACTACTCTGGGGCTCCAC |
| Enhancer3-sgRNA5-hU6-S | CACCGATGATGTTGCCACATGCAAG |
| Enhancer3-sgRNA5-hU6-AS | AAACCTTGCATGTGGCAACATCATC |
| Enhancer3-sgRNA6-hU6-S | CACCGATACAGCTGTTTTAGTGGTG |
| Enhancer3-sgRNA6-hU6-AS | AAACCACCACTAAAACAGCTGTATC |
| Enhancer3-sgRNA7-hU6-S | CACCGCTGGAGCCTAATCTTTCAGT |
| Enhancer3-sgRNA7-hU6-AS | AAACACTGAAAGATTAGGCTCCAGC |
| Enhancer3-sgRNA8-hU6-S | CACCGGTCTGGTTTGCAGGTAAAAT |
| Enhancer3-sgRNA8-hU6-AS | AAACATTTTACCTGCAAACCAGACC |
| Enhancer3-sgRNA9-hU6-S | CACCGCTCTTAAGCCTCCTGGAATA |
| Enhancer3-sgRNA9-hU6-AS | AAACTATTCCAGGAGGCTTAAGAGC |
| Enhancer3-sgRNA10-hU6-S | CACCGGGAAGAATACCTAAGGAGGC |
| Enhancer3-sgRNA10-hU6-AS | AAACGCCTCCTTAGGTATTCTTCCC |
| Enhancer3-sgRNA11-hU6-S | CACCGGAGGTCATCTAAGGTGAAAA |
| Enhancer3-sgRNA11-hU6-AS | AAACTTTTCACCTTAGATGACCTCC |
| Enhancer3-sgRNA12-hU6-S | CACCGAGAGTGAGAGCCCCGAGGGG |
| Enhancer3-sgRNA12-hU6-AS | AAACCCCCTCGGGGCTCTCACTCTC |
| Enhancer3-sgRNA13-hU6-S | CACCGAAAGGGAGTGGTAGGTTGGC |
| Enhancer3-sgRNA13-hU6-AS | AAACGCCAACCTACCACTCCCTTTC |
| Enhancer3-sgRNA14-hU6-S | CACCGCAATCTGCAACACAGAGATT |
| Enhancer3-sgRNA14-hU6-AS | AAACAATCTCTGTGTTGCAGATTGC |
| Enhancer3-sgRNA15-hU6-S | CACCGTGTTCATGATCAAATAGAGA |
| Enhancer3-sgRNA15-hU6-AS | AAACTCTCTATTTGATCATGAACAC |
| Enhancer3-sgRNA16-hU6-S | CACCGAGACTGCACAGTGAGGTAGA |
| Enhancer3-sgRNA16-hU6-AS | AAACTCTACCTCACTGTGCAGTCTC |
| promoter-sgRNA1-hU6-S | CACCGCGCGGCGCCTTTATACCCAG |
| promoter-sgRNA1-hU6-AS | AAACCTGGGTATAAAGGCGCCGCGC |
| promoter-sgRNA2-hU6-S | CACCGCACTCGCCGCTGGCAAGTTT |
| promoter-sgRNA2-hU6-AS | AAACAAACTTGCCAGCGGCGAGTGC |
| promoter-sgRNA3-hU6-S | CACCGTTAGTGAGCGCCGAGCCCGC |
| promoter-sgRNA3-hU6-AS | AAACGCGGGCTCGGCGCTCACTAAC |
| promoter-sgRNA4-hU6-S | CACCGGCGGTGGAGTCACTCGCCGC |
| promoter-sgRNA4-hU6-AS | AAACGCGGCGAGTGACTCCACCGCC |
| promoter-sgRNA5-hU6-S | CACCGAGCGCCCTACACCGCCCTCA |
| promoter-sgRNA5-hU6-AS | AAACTGAGGGCGGTGTAGGGCGCTC |
| promoter-sgRNA6-hU6-S | CACCGGACCCTGGGCCGGCGACTCG |
| promoter-sgRNA6-hU6-AS | AAACCGAGTCGCCGGCCCAGGGTCC |
| promoter-sgRNA7-hU6-S | CACCGAACCTCACGTGGACAGGCGA |
| promoter-sgRNA7-hU6-AS | AAACTCGCCTGTCCACGTGAGGTTC |
| promoter-sgRNA8-hU6-S | CACCGTCTCTGAACGCTCACTTCCG |
| promoter-sgRNA8-hU6-AS | AAACCGGAAGTGAGCGTTCAGAGAC |
| promoter-sgRNA9-hU6-S | CACCGGGCCCCCTGAGGGCGGTGTA |
| promoter-sgRNA9-hU6-AS | AAACTACACCGCCCTCAGGGGGCCC |
| promoter-sgRNA10-hU6-S | CACCGGCGCAGGCAGAAGTCACCGC |
| promoter-sgRNA10-hU6-AS | AAACGCGGTGACTTCTGCCTGCGCC |
| promoter-sgRNA11-hU6-S | CACCGGCCTCGGATGCGCAGACCCT |
| promoter-sgRNA11-hU6-AS | AAACAGGGTCTGCGCATCCGAGGCC |
| promoter-sgRNA12-hU6-S | CACCGGAGCAGGGTCCCCGAGTCGC |
| promoter-sgRNA12-hU6-AS | AAACGCGACTCGGGGACCCTGCTCC |
| promoter-sgRNA13-hU6-S | CACCGCTCTTAGCTAAAAATGACGT |
| promoter-sgRNA13-hU6-AS | AAACACGTCATTTTTAGCTAAGAGC |
| promoter-sgRNA14-hU6-S | CACCGCGCCCTCAGGGGGCCCTCGC |
| promoter-sgRNA14-hU6-AS | AAACGCGAGGGCCCCCTGAGGGCGC |
| promoter-sgRNA15-hU6-S | CACCGACTGGCAAGAGACGGAGTCC |
| promoter-sgRNA15-hU6-AS | AAACGGACTCCGTCTCTTGCCAGTC |
| promoter-sgRNA16-hU6-S | CACCGGGGCACTGGCATCCCGGCCG |
| promoter-sgRNA16-hU6-AS | AAACCGGCCGGGATGCCAGTGCCCC |
| promoter-sgRNA17-hU6-S | CACCGAAACTCACACAGCCCACCGC |
| promoter-sgRNA17-hU6-AS | AAACGCGGTGGGCTGTGTGAGTTTC |
| promoter-sgRNA18-hU6-S | CACCGGACTCCACCGCCCGGAGCAG |
| promoter-sgRNA18-hU6-AS | AAACCTGCTCCGGGCGGTGGAGTCC |
| promoter-sgRNA19-hU6-S | CACCGCGGCCGGGGGGAGCCCGCGA |
| promoter-sgRNA19-hU6-AS | AAACTCGCGGGCTCCCCCCGGCCGC |
| promoter-sgRNA20-hU6-S | CACCGTCCTGCACCGCTGCTCCGGG |
| promoter-sgRNA20-hU6-AS | AAACCCCGGAGCAGCGGTGCAGGAC |
| promoter-sgRNA21-hU6-S | CACCGGGGGTCTAAAGCAGTATGAA |
| promoter-sgRNA21-hU6-AS | AAACTTCATACTGCTTTAGACCCCC |
| promoter-sgRNA22-hU6-S | CACCGCACAGCCCACCGCAGGCAAC |
| promoter-sgRNA22-hU6-AS | AAACGTTGCCTGCGGTGGGCTGTGC |
| promoter-sgRNA23-hU6-S | CACCGGGGGAAAGGGCGCGCGGCCT |
| promoter-sgRNA23-hU6-AS | AAACAGGCCGCGCGCCCTTTCCCCC |
| promoter-sgRNA24-hU6-S | CACCGGAGCAGGCGCGCGTGGCGCG |
| promoter-sgRNA24-hU6-AS | AAACCGCGCCACGCGCGCCTGCTCC |
| promoter-sgRNA25-hU6-S | CACCGCAGCCGGGGCGCAGAGTGCG |
| promoter-sgRNA25-hU6-AS | AAACCGCACTCTGCGCCCCGGCTGC |
| promoter-sgRNA26-hU6-S | CACCGCAGTTCCAGTTGCCTGCGGT |
| promoter-sgRNA26-hU6-AS | AAACACCGCAGGCAACTGGAACTGC |
| promoter-sgRNA27-hU6-S | CACCGGCGGGGCCCGGAGGAGCCGT |
| promoter-sgRNA27-hU6-AS | AAACACGGCTCCTCCGGGCCCCGCC |
| promoter-sgRNA28-hU6-S | CACCGTCAGTTCCAGTTGCCTGCGG |
| promoter-sgRNA28-hU6-AS | AAACCCGCAGGCAACTGGAACTGAC |
| promoter-sgRNA29-hU6-S | CACCGGGCAACTGGAACTGAAACCC |
| promoter-sgRNA29-hU6-AS | AAACGGGTTTCAGTTCCAGTTGCCC |
| DPP4 KO1 | CTTAGAATACAACTACGTGA |
| DPP4 KO2 | CTAACCAATTTATGACCCAC |

| Human DPP4 CRISPR Guide RNAs KO1 and KO2 constructs were ordered from GenScript |
| --- |

**Table S7.2. shRNA sequences.**

| **Name** | **Sequences** |
| --- | --- |

| *Dpp4* shRNA1 (KD1) | ATAGCTCTCCATAGCTTATTT |
| --- | --- |
| *Dpp4* shRNA2 (KD2) | CCAAGAAATATCCTCTACTAT |
| PU.1 shRNA1 (PU.1 KD1) | GCAGATGCACGTCCTCGATAC |
| PU.1 shRNA5 (PU.1 KD2) | CAAGAAGAAGATCCGCCTGTA |

| shRNA bacteria clones were ordered from Millipore Sigma |
| --- |
